# Transcriptome, proteome, and protein synthesis within the intracellular cytomatrix

**DOI:** 10.1016/j.isci.2023.105965

**Published:** 2023-01-13

**Authors:** Tattym E. Shaiken, Sandra L. Grimm, Mohamad Siam, Amanda Williams, Abdol-Hossein Rezaeian, Daniel Kraushaar, Emily Ricco, Matthew J. Robertson, Cristian Coarfa, Antrix Jain, Anna Malovannaya, Fabio Stossi, Antone R. Opekun, Alyssa P. Price, Julien Dubrulle

**Affiliations:** 1 Department of Medicine-Gastroenterology and Hepatology Section, Michael E DeBakey Veteran’s Affairs Medical Center, Baylor College of Medicine, Houston, TX 77030, USA; 2 PeriNuc Labs, University of Houston Technology Bridge, Houston, TX 77023, USA; 3 Advanced Technology Cores, Baylor College of Medicine, Houston, TX 77030, USA; 4 Department of Drug Discovery and Biomedical Sciences, University of South Carolina, Columbia, SC 29208, USA; 5 Department of Molecular and Cellular Biology, Baylor College of Medicine, Houston, TX 77030, USA; 6 Department of Pediatrics, Baylor College of Medicine, Houston, TX 77030, USA

**Keywords:** Molecular biology, Cell biology, Proteomics, Transcriptomics

## Abstract

Despite the knowledge that protein translation and various metabolic reactions that create and sustain cellular life occur in the cytoplasm, the structural organization within the cytoplasm remains unclear. Recent models indicate that cytoplasm contains viscous fluid and elastic solid phases. We separated these viscous fluid and solid elastic compartments, which we call the cytosol and cytomatrix, respectively. The distinctive composition of the cytomatrix included structural proteins, ribosomes, and metabolome enzymes. High-throughput analysis revealed unique biosynthetic pathways within the cytomatrix. Enrichment of biosynthetic pathways in the cytomatrix indicated the presence of immobilized biocatalysis. Enzymatic immobilization and segregation can surmount spatial impediments, and the local pathway segregation may form cytoplasmic organelles. Protein translation was reprogrammed within the cytomatrix under the restriction of protein synthesis by drug treatment. The cytosol and cytomatrix are an elaborately interconnected network that promotes operational flexibility in healthy cells and the survival of malignant cells.

## Highlights

• The cytoplasm is a two-phase system consisting of viscous fluid and elastic solid
• The elastic solid can be separated, from the viscous fluid, as the cytomatrix
• The cytomatrix immobilizes catalytic complexes for efficient biocatalysis
• Immobilization and segregation of catalytic complexes and proteome can form organelles

## Graphical abstract

**Figure:**
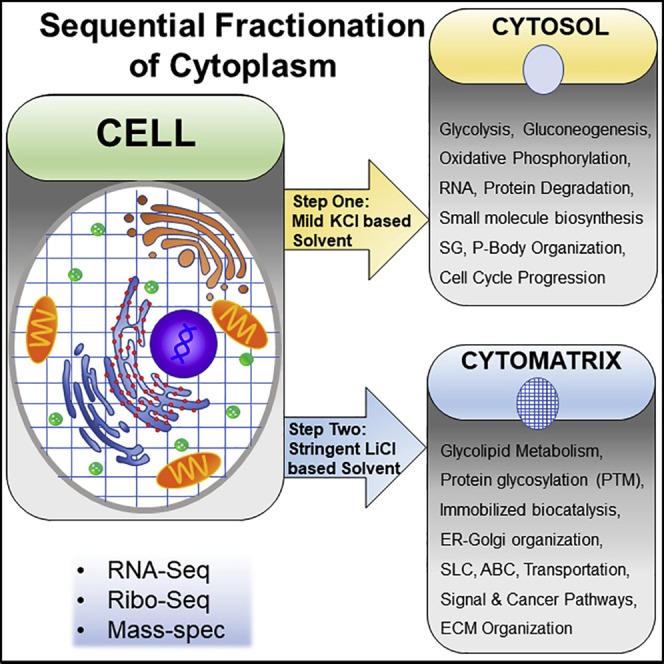

For a Figure360 author presentation of this figure, see https://doi.org/10.1016/j.isci.2023.105965.

## Introduction

The central problem of cell biology is the organization of biochemical reactions and organelles in the cytoplasm and how organelles need to be assembled in the cytoplasmic space after cell division or depending on cell conditions. Since the 19^th^ century, scientists have made substantial efforts to understand the physical and chemical properties of the cytoplasm, and debated the nature of the cytoplasm.^1^ Still, the structural organization of the cytoplasm remains a contentious topic. For the past hundred years since its inception, the concept that cell cytoplasm is a gel-like substance has been broadly acknowledged.^2^ However, the mechanisms of chemical reaction in the cell are routinely explained based on the idea of free diffusion, as if the cells were an aqueous solution.^3^ The cytoplasm is a dynamic, crowded, and heterogeneous environment, but this concept gives little help in understanding the cytoplasmic structure and organization. The cytoplasm is involved in protein synthesis, energy generation, nutrient processing, transport, and metabolism, which also contributes to nuclear processes in response to external stimuli or stressors. Capable of managing a myriad of chemical reactions, the structure of the cytoplasm is complex, with enzymes and metabolic pathways organized into clusters,^4^ with little space available for soluble species to roam.^5^

Current theories suggest that the cytomatrix is an elastic solid within which intermediate filaments anchor organelles—interdependent structures that communicate through contact sites^6^—against fluctuating cytosolic forces.^7^ Further, the structure of a large portion of the intracellular water is dictated by interactions with the cell ultrastructure, driving changes in cytoplasmic states from a viscous fluid to an elastic solid.^8^

The cytomatrix (CMX) is thought to be a proteinaceous matrix that forms a network with the cytoskeleton to integrate metabolic pathways.^1^ The CMX is traversed by channels and delimited by microtubules throughout which it associates with organelles, ER ribosomes, and metabolic protein complexes.^9^ Structural proteins are also an integral part of the cytoplasmic matrix, wherein the nucleus and cytoplasm are connected through the Linker of Nucleoskeleton and Cytoskeleton (LINC) complex.^10^^,^^11^^,^^12^ The integrity of protein structure and macromolecular complexes within the CMX are supported by the ionic microenvironment that maintains a thermodynamically favored stable equilibrium.^13^ The potassium ion is the principal intracellular electrolyte necessary for the function of all living cells and the most abundant intracellular cation^1^ involved in protein synthesis. Potassium ions are involved in the stabilization of main functional ligands such as mRNA and transfer RNAs, as well as ribosomal RNAs and ribosomal proteins.^14^

Mounting evidence suggests that among the solid macromolecular matrix exists a viscous liquid, consisting of both water-soluble proteins and biopolymers along with lipid membrane-associated and lipid-wrapped protein cargo. Thus, from a physicochemical standpoint, the cytoplasm could be considered a two-phase system consisting of a viscous fluid and an elastic solid. To date, however, biophysical analysis and microscopic techniques have not distinguished the viscous fluid and elastic solid elements. Moreover, the cytoplasm and cytomatrix are often defined as equal terms. Hence, the question of how to isolate the viscous fluid phase (i.e., cytosol) from the elastic solids component (CMX) remains.

The only way to reveal the elastic solid matrix (i.e., CMX) is to chemically remove the viscous fluid (i.e., cytosol). Removing the viscous fluid may not compromise the integrity of the CMX and connections with the nucleus through the LINC complex under certain conditions. Organelles differ in their molecular structure, particularly in the composition of proteins and other biopolymers that dictate their specific physicochemical characteristics. Subcellular fractionation methods are based on the ability of cell organelles to withstand specific salt and detergent concentrations. It has been shown that the inner nuclear membrane (INM) is resistant to high salt and detergent concentrations, and that the nuclear pore complex (NPC) does not require an intact outer nuclear membrane.^15^ However, it took the work of several generations of cell biologists to determine that treating cells with the nonionic detergents such as Triton X-100 does not cause spilling of nuclear contents due to the discovery that the INM and NPC proteins are associated with the nuclear lamina.^16^ To this end, it was shown that an approximately 15-nm-thick layer lamina with attached NPCs encircle the entire nucleus, but does not extend into its interior.^17^ Prior studies have also shown that the Golgi matrix is a detergent- and salt-resistant complex that is easily isolated from purified rat liver Golgi.^18^

As first reported in 1888, the effects of inorganic salts on proteins and macromolecules in aqueous solutions generally follow a Hofmeister series.^19^^,^^20^ Specifically, the effects of cations are important for protein folding, protein−protein interactions, and protein aggregation. Strongly hydrated cations such as lithium (Li^+^) are the most effective for salting proteins into solution, whereas weakly hydrated cations lead to salting-out behavior. When the interaction of different cations with the model compound, butyramide, was studied, it was shown to have statistically significant binding amid oxygen for Li^+^ but weak binding for Ca^++^ and Mg^++^ and exclusion for Na^+^ and K^+^.^21^

Since it has been established that Li^+^ interacts with peptide groups,^22^ lithium salts have been widely investigated as a member of the Hofmeister series. In model polymers such as oligoglycines, which are insoluble in water, lithium salts increase the solubility of polyglycine, a property that likely contributes to the denaturing ability of lithium salts.^23^ For alkali cations, the bond dissociation energies for Li-ions are the highest.^24^ As well, direct protein-ion interactions are responsible for the Hofmeister effects of ions on protein stability. Further, at low ion concentrations (<100-200 mM), Li-ions specifically interact with biopolymers.^25^ Similarly, it has been shown that Li cations exhibit a strong salting-in effect through improved solvation of the amide carbonyl group.^26^ The charge-dense Li ions dissolve hydrophobic molecules. Li cations, however, can also interact with a variety of functional groups, suggesting that the apparent Li-induced lowering of hydrophobicity may be a result of specific interactions between the functional groups and Li ions.

Recently, we used LiCl salt to separate a nuclear-associated fraction from the cytosolic and nuclear fractions in an embryonic fibroblast and two cancer cell lines, an approach that distinguishes our method from traditional cell fractionation methods.^27^ Our current work enabled us to define the nuclear-associated cytoplasmic fraction without the cytosol as the cytomatrix. In our extraction protocol, we used Hofmeister’s series of ion properties and developed an equation to calculate ionic concentrations for separating the CMX and the cytosol. The cytosol was dissolved using a detergent-containing isotonic buffer that does not cause osmotic stress due to the same osmotic pressure. The CMX was then separated from the nuclear fraction with the LiCl salt-containing stringent isotonic buffer, and the extract was solubilized with the DOX/Triton X-100. Hence, we distinguish the cytomatrix as a detergent and salt-resistant composite, the elastic solid phase of cytoplasm. In contrast, we define the cytosol as a detergent-sensitive, dynamic liquid phase of the cytoplasm that can be isolated using nonionic detergents under the physiological osmotic conditions.

Here, we determined compartmentalization of transcriptome, proteome, metabolome, and protein synthesis within the CMX and found that CMX-associated ribosomes are distinct in mRNA transcript assortment. Enrichment of biosynthetic pathways indicated the presence of immobilized biocatalysis within the cytomatrix. Efficient biocatalysis achieved by segregating and immobilizing the metabolome enzymes can overcome spatial impediments to biochemical reactions, which implies the CMX involvement in cytoplasmic organelle formation and assembly. Accordingly, the dynamics of the cytoplasmic organelles may depend on the organization of the CMX. High-throughput analysis and conventional methods revealed different responses from the cytosol and CMX during times of drug-induced protein deficiency. The reprogrammed protein translation within the cytomatrix shows operational flexibility of the cytoplasmic phases that promote cell survival under extreme conditions.

## Results

### The cytoplasm is a two-phase system

Our objective was to isolate the CMX and compare this elastic solid phase of the cytoplasm with the cytosolic viscous fluid, or liquid, phase of the cytoplasm. To this end, our two-step strategy for isolating the CMX (Figure S1A) included the initial separation of the dynamic, viscous fluid fraction of the cytoplasm using a nonionic detergent. To avoid osmotic stress and keep the CMX intact, we used a physiological concentration of potassium ions and a minimal solvent volume. The first dissolved cellular material was the cytoplasmic solution, otherwise known as the cytosol. In the second step, we used a stringent buffered solvent, containing lithium ions, to separate the CMX from the nucleus. Thus, using two buffers we separated the cytosolic, CMX, and nuclear fractions. Two different nonionic detergents (NP-40 and Triton X-100) were used to solubilize the cytoplasmic material. To prevent rupturing the basic structure of organelles, the cytosol and CMX were extracted under physiologically osmotic conditions. During the first step of the separation technique, the mild buffer extracted only the cytosol while the CMX was pelleted with the nucleus as a non-dissolved solid phase (Figure S1A). The CMX was subsequently isolated from the nucleus using a second, stringent solvent.

We found that the viscous fluid dynamic phase of the cytoplasm was salt and detergent sensitive and could be extracted using the nonionic detergent NP-40 or its analog. To prevent damage to the elastic solid cytomatrix, we used potassium salt, which replicates the natural ionic environment of the cell. Contrary to the cytosol, the CMX was salt and detergent resistant but sensitive to lithium salt. Thus, we used LiCl to dissociate the CMX from the nucleus and to break up the macromolecular complexes and Triton X-100/DOX for solubilization. Due to the strong salting-in or dissolving effect of Li cations, our method required a precise calculation of the lithium salt concentration to ensure that dissociating the CMX did not rupture the nucleus. The KCl concentration was also calculated to avoid the osmotic stress to keep the CMX intact during the cyto-solution (cytosol) extraction. A saline solution containing 0.9% sodium chloride (154 mM NaCl) was used as the standard to calculate the LiCl and KCl salt concentrations in our solvents.

In aqueous solutions, salts are dissociated to ions and ions are surrounded by a hydration or solvation shell. The relative ionic radius^28^ is a solvation shell of ions in solution that surround proteins or protein complexes in the cell. Because the ionic ratio of a protein solution depends on the relative ionic radius, we developed an equation to calculate the concentration of ions to replace sodium ions in the standard isotonic solution based on the relative ionic radius (Figure S1B). We found that solutions containing 120 mM KCl and 200 mM LiCl do not cause osmotic pressure. Further, our calculated concentration was similar to the concentration values obtained experimentally for erythrocyte hemolysis,^29^ in which the isotonic molarity of the LiCl solution was 0.189 M. Experimentally, it was confirmed that 0.2 M LiCl salt does not rupture the nucleus, but if this concentration were to be exceeded, the nuclear contents would spill out.

The solubilization of lipid bilayers using detergents is a slow process at 4°C,^30^ and can involve several steps. The sequence of events that must occur include: 1) relatively rapid penetration of detergent monomers into the outer lipid monolayer; 2) transmembrane equilibration of detergent monomers between the two monolayers; 3) saturation of the lipid bilayer by detergents and consequent permeabilization of the membrane; and 4) transition of the entire lipid bilayer to thread-like mixed micelles.^31^^,^^32^ Mixing the collected cells via rotation with Buffer A for 30 min provided time for the micelles, which envelop lipids and proteins immersed in lipid bilayers, to form (Figure S1C). Therefore, water-soluble extracts of the cytosolic cellular contents, and lipids of the ER, plasma membrane (PM), and outer nuclear membrane (ONM), would be contained within the suspended micelles and dispersed in a liquid colloid. Hence, the formation and stabilization of the micelles prevent post-lysis reassortment.^33^

To validate the fractionation method, we compared our protein extraction procedure with a widely used cell lysis buffer from Cell Signaling Technology (cat# 9803). The percent of proteins in each fraction was determined by dividing the concentration of proteins found in each fraction by the total protein concentration. Approximately 70% of the total colorectal cancer cell (HCT-15) proteins were detected in the cytosol. Further, the remaining proteins were roughly equally distributed between the nuclear and CMX fractions (Figure S1D). Our results were consistent with previous findings in four other cancer and normal cell lines.^27^ Notably, the concentration of proteins in the cytosol fraction was approximately 10% greater using the conventional cell lysis buffer containing 150 mM NaCl, indicating that CMX protein recovery was divided between the cytosolic and the nuclear fractions (Figure S1E).

**Figure 1:**
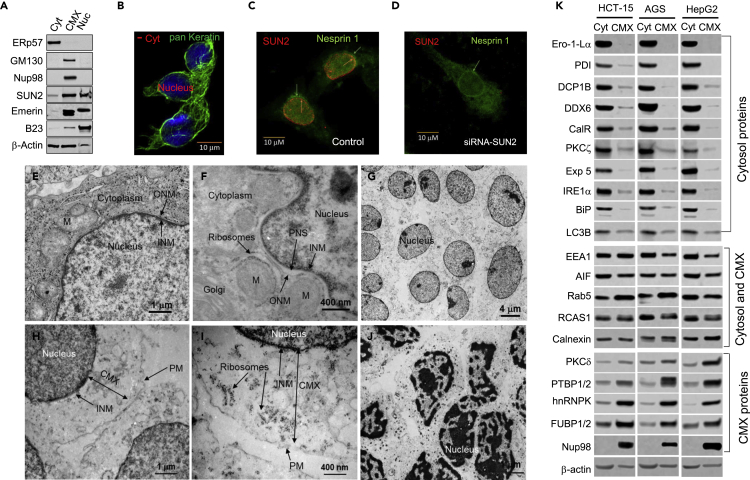
Illustration of cytomatrix isolation The cytosol and cytomatrix were sequentially extracted from HCT-15 cells. The cytosol was extracted with nonionic detergent containing mild solvent, whereas the cytomatrix was separated from the nuclear fraction using a buffered, stringent solvent. (A) Immunoblots of marker proteins in fractions. (B) Immunofluorescence image of keratin localization in cytosol-removed cells. (C) Immunofluorescence images of SUN2 (red) and Nesprin-1 (green) localization in HeLa cells. (D) Nesprin-1 is predominantly localized around the nucleus in SUN2 knockdown cells. (E-J) TEM micrographs of HCT-15 cells. Ultrastructure of the nuclear periphery of (E and F) an intact cell and (G-I) a cytosol-removed cell. (J) cytosol and cytomatrix removed core nuclei. (K) Protein expression in the cytosol versus cytomatrix of three cell lines. The cytosol and cytomatrix were run side by side, but bands were cut based on cell line to show differences in protein expression in corresponding compartments. Western blots were run in the same gel. The three cell lines show consistent results for predominantly cytosolic proteins (upper panel), equally distributed proteins (middle panel), and predominantly cytomatrix proteins (bottom panel). Cyt – cytosol, CMX – cytomatrix, M− mitochondria, INM – inner nuclear membrane, ONM – outer nuclear membranes, PM – plasma membrane carcass, PNS – perinuclear space.

To distinguish the cytomatrix from the cytosol we studied marker proteins of the corresponding fractions. Western blot analysis of proteins in the cytosolic, CMX, and nuclear fractions from HCT-15 colon cancer cells shows the differential distribution of well-known cellular markers (Figure 1A). The nuclear envelope proteins SUN2 and emerin were found in the CMX and nuclear fractions. With a lower molecular weight, emerin was spotted only in the CMX. Conversely, the nucleolar protein B23 was found predominantly in the nuclear fraction. The nuclear pore complex protein Nup 98 was detected exclusively in the CMX, suggesting a potential new marker for this compartment. The NPC attached to the INM^17^ cannot be extracted to the cytosol by the mild isotonic first buffer, even if the ONM dissolves.

The GM130 protein was previously identified in the Golgi matrix fraction as an inter-cisternal structural component.^34^ Because it is known that the Golgi matrix is detergent and salt resistant,^18^ we did not expect GM130^35^ to be detected in the cytosol fraction. To this end, we found that GM130 was the most abundant protein detected within the CMX fraction, indicating that the Golgi matrix could be an integral part of the CMX. Cellular markers also were visualized by immunofluorescence. After cytosol extraction, keratin filaments and the LINC complex proteins (i.e., SUN2 and Nesprin-1) were localized to the nuclear surface (Figures 1B-1D). The nucleus is well protected by the LINC complex proteins and keratin, which allowed us to separate the core nucleus from the CMX. A previous study demonstrated a keratin cage formation in cultured monolayers of living cells by time-lapse imaging^36^ that supports our finding of the keratin encapsulation of the nucleus.

The nuclear membrane is resistant to high salts and detergents due to nuclear encapsulation with filamentous proteins.^15^ Finally, in addition to the LINC complex, approximately 200 proteins protect the INM,^37^ which prevented a rupture of the nucleus and allowed us to separate the CMX from the nucleus with the second Li^+^ containing stringent buffer.

Transmission electron microscopy (TEM) was used to visualize the outcome of fractionation at the ultrastructural level. The resulting micrographs revealed that intact HCT-15 cells had a well-defined perinuclear space, nuclear envelope, Golgi, mitochondria, and ribosomes (Figures 1E and 1F). After removing the cytosol, the nucleus had a higher electron density, likely due to cytoplasmic lipid deprivation that contrasted the nucleus on the cytoplasmic background (Figures 1G-1I). The INM, the carcass of the PM and ribosomes were clearly visible. The outer nuclear membrane was dissolved. Notably, the buffer used to extract the CMX from the nucleus caused chromatin condensation (Figure 1J). Our results indicate that the CMX buffer partially dissolved the LINC complex bridge,^38^ separating the CMX from the nuclear matrix. Thus, the CMX appeared to stretch from the INM to the lipid-depleted carcass of the PM.

The isolation of the CMX was validated in two additional digestive system cell lines, AGS and HepG2 cells (Figure 1K). Calnexin (CNX) and calreticulin (CalR) are chaperons that control proper protein folding in the ER in cooperation with the BiP, ERp57, PDI, and Ero-1-Lα proteins.^39^^,^^40^ Except for CNX (Figure 1K, middle panel), which is equally distributed between the cytosol and CMX, all other members of the protein folding process were detected predominantly in the cytosol (Figures 1A and 1K).

CalR is a soluble protein that is retained in the ER lumen, whereas CNX is localized to the cytoplasm via a single-pass transmembrane domain that couples it to the outer face of the ER membrane.^39^ However, CNX can translocate to the lumen during the protein folding process, as evidenced by an equal distribution of CNX within the cytosol and CMX. The ER is composed of distinct structures that include tubules, matrices, and sheets.^41^ Further, the ER membrane proteins tubulin, reticulon, p180, and kinectin, as well as the DP1, REEP, and Climp63 proteins and the ER luminal protein calumenin-1 maintain ER sheet morphology and shape.^42^^,^^43^^,^^44^ Besides well-visualized lipid membranes, the ER is an organized proteinaceous net, the hidden morphology of which can be revealed by the cryo-ET.^45^ We did not expect our mild isotonic buffer to rupture the proteinaceous carcass of the ER, but rather to extract the transporting luminal proteins and the lipid membrane. To this end, we detected ER luminal proteins (Figure 1K) and the dynamic lipid layers in the cytosol following extraction, as evidenced by TEM, the depletion of lipids after cytosol extraction in the CMX (Figures 1G and 1H).

Factors associated with RNA degradation, including LC3B, DCP1B, DDX6, Exportin 5, and IRE1α were also detected primarily in the cytosol,^46^^,^^47^ whereas proteins related to endosome biogenesis, such as EEA1, Rab5, RCAS, and AIF,^48^^,^^49^ were equally distributed between the cytosol and CMX. Of the additional proteins tested, the calcium-insensitive isoform of PKC delta^50^ and the mRNA binding proteins FUBP1/2, hnRNPK, and PTBP1/2^51^^,^^52^^,^^53^ were enriched in the CMX.

### Assessment of protein synthesis in the cytosol and the cytomatrix

To address differential mRNA translation in the cytosol and CMX, we needed a method that statistically detects ribosome biogenesis and protein biosynthesis. For ribosome biogenesis, ribosomal proteins were an obvious choice; moreover, ribosomal protein L7a (rpL7a) was a large subunit protein. The rpL7a belongs to the “druggable genome” protein family, the genome-encoding proteins that small molecules can regulate. Because the mRNA translation is controlled by 80S formation and 60S availability^54^ and the nucleolar hypertrophy is an indicator of ribosome biogenesis,^55^ the rpL7a was an appropriate choice. In addition, rpL7a is a highly conserved ribosome protein localized on the surface of the 60S^56^^,^^57^ and contains two distinct RNA binding domains^58^ and an FKBP25 binding domain,^59^ suggesting that rpL7a may play an essential role in 60S subunit biogenesis. Notably, the localization of rpL7a on the surface of the 60S ribosomal subunit enables the use of rpL7a as a readout for the ribosomal large subunit biogenesis and protein expression.

We preliminarily tested the effects of several compounds that suppress diverse signaling pathways: pp242, Torin 1 (mTOR inhibitors), PF-4708671 (RSK), Bio (GSK-3), Rotenone (mitochondrial electron transport), MG132 (26S proteasome), and Geldanamycin (Hsp90) on the ribosomal protein translation (Figures S2A-S2J). We expressed GFP-tagged rpL7a in HCT-15 cells and monitored the resulting fluorescence in living cells. Although the compounds inhibit different signaling pathways, all compounds upregulated rpL7a translation. Additionally, rpL7a displayed the perinuclear localization and accumulation in the nucleus. rpL7a contains a domain II (residues 52-100) that directs nuclear and nucleolar localization.^60^ The increase of rpL7a biosynthesis in the cytoplasm, in response to chemical agents, has likely partially delayed the nuclear entry of an excessive number of proteins at the nuclear envelope, presenting itself as a bright rim around the nucleus (Figures S2C-S2J). Consequently, the intensification of the 60S biogenesis occurring in the nucleus, was revealed as nucleolar hypertrophy, due to an excessive accumulation of rpL7a. High-throughput tests confirmed the number of nucleoli and average nucleoli size was significantly increased in treated cells (Figures S2K-S2M). Since all compounds elicited similar results, we selected the mTOR kinase inhibitor (mTOR-KI), pp242,^61^ for further analysis.

**Figure 2:**
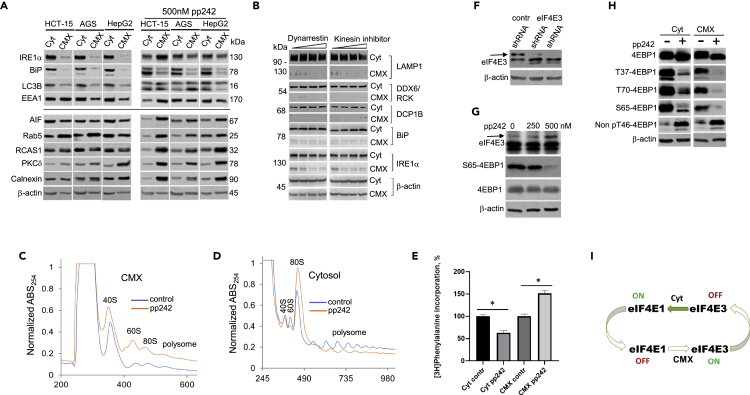
Analysis of protein synthesis in the cytosol and cytomatrix (A) Protein distribution in the cytosol and cytomatrix of three cell lines under the normal growth conditions (left panel) and 500 nM of pp242 treatment for 24 h (right panel). (B) The effect of increasing concentration of motor protein inhibitors on protein distribution in the cytomatrix and cytosol of HCT-15 cells. Inhibitors of the dynein and kinesin proteins showed no effect on protein distribution. To minimize mixing during loading, the equal amounts of samples were run separately. Side by side running gel data for the same samples are presented in Figures S3A and S3B. (C and D) Polysome profile of the cytomatrix and cytosol of HCT-15 cells. (E) The incorporation of radiolabeled L-[^3^H]-phenylalanine into proteins of the cytosol, and the CMX in untreated and pp242-treated HCT-15 cells. L-[^3^H]-phenylalanine was added, and cells were incubated for 1 h. ∗p-value of one-way ANOVA, p < 0.0001. Data are given as means ± SE, n = 5. (F) eIF4E3 is localized in the cytomatrix of HCT-15 cells. (G) The pp242 increased eIF4E3 protein expression in the CMX of HCT-15 cells. (H) eIF4E-BP1 phosphorylation in the cytosol and cytomatrix with and without pp242 treatment of HCT-15 cells. (I) Hypothetical diagram of inverse relationship of the eIF4E1 and eIF4E3. eIF4E – eukaryotic initiation factor 4E. BP1 – eIF4E binding protein 1.

mTOR-KI inhibits mTOR-dependent protein translation and directly affects autophagy and PI3K-Akt signaling.^62^^,^^63^ Treating cell lines with pp242 shifted the localization of several proteins toward the CMX and induced posttranslational modifications (Figure 2A, right panel). Determining whether proteins are translated in the CMX or translocated is central to characterizing CMX function. To test whether motor proteins facilitate cytosol/CMX protein translocation, HCT-15 cells were treated with dynein and kinesin inhibitors, and proteins in the cytosol and CMX were compared (Figures S3A and S3B). No significant protein translocation from the cytosol to the CMX or vice versa following treatment with Dynarrestin and K858 were detected. Proteins involved in the mRNA and protein recycling pathways, including LAMP1,^64^ DDX6/RCK,^65^ DCP1B,^66^ IRE1α,^47^ and BiP,^67^ were exclusively localized within the cytosol (Figure 2B).

mTOR inhibitors remove ribosomes from polysomes and prevent polysome aggregation.^68^ We used polysome profiling to compare differences of the cytosol and CMX under pp242 treated conditions and normal growth conditions in HCT-15 cell lines. After inhibitor treatment, the polysome profiles in the cytosol and CMX were reversed (Figures 2C and 2D). In the CMX, polysomes and large ribosomal subunit levels increased in response to inhibitor treatment. In the cytosol, the 60S and polysome levels decreased and monosome (80S) levels increased in response to pp242 treatment. The ratio of the small subunit 18S rRNA in the cytosol and CMX, determined by RT-PCR, was 1.8 to 1 (Figure S3C). To address the 40S subunit dynamics at the normal growth condition and pp242 treatment, the mRNA of small ribosomal protein rpS6 level was monitored using single-molecule fluorescence *in situ* hybridization (Figure S3D). Even after pp242 treatment for 2 h, the cells had higher levels of *rpS6* mRNA signal. A steady state abundance of rpL7a and the rpS6 mRNA expression evidenced the rise of ribosome biogenesis under the pp242 treatment. However, these results do not clarify the translated spectrum of the proteins.

To further address observed protein synthesis dynamics, we quantified protein synthesis using a radiolabeled amino acid to detect newly synthesized proteins. Incorporating L-[^3^H] phenylalanine into proteins revealed that the mTOR inhibitor pp242 decreased *de novo* protein synthesis in the cytosol by 20%, which aligns with prior reports of global protein translation suppression.^61^^,^^69^ However, the incorporation of labeled amino acids increased in CMX proteins by 50% (Figure 2E). The latter result indicated that *de novo* protein synthesis increased in the CMX when global protein translation was suppressed in the cytosol, the latter of which also was detected by polysome profiling (Figures 2C and 2D). Thus, using six different methods—western blotting, polysome profiling, GFP-tagged rpL7a immunofluorescent technique, high-throughput nucleolar hypertrophy test, *in situ* hybridization of rpS6 mRNA, and the incorporation of radiolabeled amino acids during protein synthesis—we showed that protein biosynthesis could differ in the CMX and the cytosol following pp242 treatment. These data may indicate an unknown cell survival mechanism based on the translational capabilities found in the CMX.

Next, we sought to determine whether the increased CMX protein translation following the pp242 treatment could be explained by an mTOR-independent pathway. The switch from eIF4E1-dependent to eIF4E3-dependent protein translation has been demonstrated in a few cell lines.^70^ After validating the specificity of an anti-eIF4E3 antibody (Figure 2F), we treated HCT-15 cells with increasing concentrations of pp242 to determine the effects of mTOR-KI on eIF4E3 expression. A dose-dependent increase in eIF4E3 protein levels in the CMX was observed (Figure 2G). Inhibiting 4E-BP1 phosphorylation using the same compound in the cytosol and CMX appears to be in the inverse relationship with the eIF4E3 level in the CMX (Figure 2H). Results indicate the possibility of activating other protein synthesis regulatory pathways in CMX (Figure 2I).

### Mass-spectrometry-based proteome profiling of the cytomatrix

Large-scale protein identification with mass spectrometry-based proteomics is an appropriate way of categorizing the proteome content of the CMX. Proteins that could form the structural foundation of the CMX, the cytoskeleton-associated proteins, actins, keratins, tubulins, and other cellular microfibrils were identified (Table 1). The Golgi, ER, nuclear pore complex proteins, and nuclear and plasma membrane proteins also were enriched in CMX. Thus, mass spectrometry data confirmed immunoblotting results and supported the notion that the CMX is enriched with the Golgi and ER matrices proteome. Enzymes and substrates of protein glycosylation, prenylation, and protein-lipid modification are the types of posttranslational modification (PTM) that were identified within CMX, confirming the presence of the ER-Golgi matrix constituency. These also happen to be the most abundant types of PTM of proteins that can promote the immobilization and segregation of protein and enzyme complexes.^71^ Extracellular matrix proteins, desmosomes, cell junction proteins, integrins, and receptors also were enriched. Interestingly, along with the normal cell signaling pathway proteins, the CMX contained a broad spectrum of proto-oncoproteins, which suggests the essential role of CMX in cancer development. A full spectrum of the CMX’s functional proteome is represented as a pie chart (Figure S4A). The CMX proteome was grouped based on the biological function of proteins (Table S1).

**Table 1:** Selected mass spectrometry based cytomatrix proteomics

	Structural proteins	Quantity	Enzymes and posttranslational protein modification	Quantity	Receptors, proto-oncogenes	Quantity
1	Cytoskeleton-associated	7	Glycosyltransferase	4	Ras family	15
2	Golgi proteins	88	Oligosaccharyltransferase	6	Src family	9
3	ER/microtubule proteins, Receptors, Ca^2+^ homeostasis	48	Mannosyl-glycoprotein beta-1,2-N-acetylglucos-aminyl transferase	7	Tumor, metastasis associated proteins	48
4	Nuclear membrane	22	Mannosyltransferase	15	BRCA associated	7
5	Nucleoporins	24	Mannosyl-oligosaccharide glucosidase	1	Retinoblastoma	9
6	PM, membrane proteins	166	Mannosidase	6	Melanoma	8
7	AHNAK/desmosome	3	Acetylgalactosaminyl transferase	7	Abl Tyr-kinase	5
8	Transmembrane proteins	61	Dolichyl-saccharide-transferase	6	MYC associated	7
9	Junction proteins	11	O-linked GlcNAc transferase	4	MYB binding protein	2
10	Matrix proteins	9	Procollagen- 2-oxoGl-dioxygenase	3	Cbl proto-oncogene	4
11	Cell adhesion	12	Collagen b-(1-O) galactosyltransferase	2	Jun proto-oncogene	2
12	Actin, actin-binding, actin-like or related	98	Polysaccharide biosynthesis domain	1	DEK proto-oncogene	1
13	Tropomyosin/myosin	29	Phosphatidylinositol transfer protein	5	Growth arrest, arrestins	6
14	Keratins, type	25	Phosphatidylinositol glycan synthesis	8	Set proto-oncogene	2
15	Tubulins	9	α-2-HS-glycoprotein	11	Erb-b2 receptors	6
16	Profilins/cofilins	4	UDP-glucose glucosyl transferase	1	Growth factors, receptors	29
17	Catenin/cadherin	5	O-methyltransferase	2	G protein binding	30
18	Pleckstrin homology	16	Serine palmitoyl-transferase, long chain	1	Inositol-PPP-receptor	3
19	Stathmins	2	Palmitoyl-protein thioesterase	5	Cell division cycle, cyclins	49
20	Lectins	7	Membrane proteins, palmitoylated	3	Apoptosis signaling	21
21	Annexins	10	N- myristoyltransferase	1	p53 tumor suppressor	4
22	Spindle proteins	13	Farnesyl-PP-synthase	1	TNF receptors	8
23	Integrins	13	Farnesyl-PP farnesyltransferase	3	Calcium signaling	19
24	Adducin	2	Protein prenyltransferase	1	Hypoxia inducible family	3
25	Vinculin	1	Isoprenylcysteine methyltransferase	1	cAMP responsive element	2

Cytosolic enzyme spectrum is comprised of protein and RNA degradation pathways, lipid, nucleotide, and amino acid metabolism, and glycolysis, which can be categorized as the nutrient and energy processing cycle of the cell (Figure S4B).

**Figure 3:**
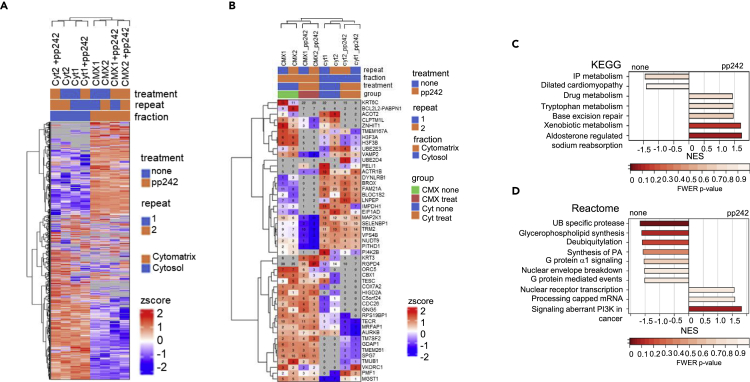
Mass spectrometry proteome analysis of the cytomatrix and cytosol of HCT-15 cells (A) Heatmap of protein expression from cytosol and cytomatrix of the control and pp242 treated HCT-15 cells. (B) Heatmap of 25 selected proteins in each differential analysis. (C and D) KEGG and Reactome pathway analyses of CMX proteome of the untreated and pp242 treated HCT-15 cells for 24 h. NES – normalized enrichment score.

A peptide area-based quantification (iBAQ) showed a relative protein level of the CMX and the cytosol proteome (Table S2). Differences in the corresponding proteomes were calculated as fold change in the CMX compared with the cytosol and vice versa, the results of which indicated an inverse relationship between the cytosolic and CMX proteome. Hierarchical clustering of the proteins detected with the respective proteomes revealed differences between the cytosol and CMX proteomes, as well as between the control and TOR-KI treated cells (Figures 3A and 3B). KEGG and Reactome pathway analyses likewise revealed the effect of mTOR-KI on the proteomes (Figures 3C and 3D).

### Differences of the cytosol and cytomatrix and correspondence of RNA-seq and Ribo-seq

RNA-seq and Ribo-seq are powerful tools that identify differentially expressed genes (DEGs). DEGs permit over-representation (or enrichment) analysis (ORA), a statistical method that determines whether genes from pre-defined sets are present more than expected. ORA of the pre-defined gene sets Hallmark (Table S3), KEGG (Table S4), Reactome (Table S5), and GOBP (Table S6) permitted analysis of specific pathway distribution within the cytosol or CMX.

To distinguish pathways of the cytosol and CMX, we prepared ribosomal footprint and mRNA libraries for sequencing from untreated and pp242-treated HCT-15 cells. Using ribosome profiling (Ribo-Seq), which is based on the deep sequencing of ribosome-protected footprints of translated mRNA,^72^ the translation differences were compared between the cytosol and the CMX. RNA-seq and Ribo-seq datasets were used to establish a transcriptome and translatome map of the cytosol and CMX, respectively. The expression of approximately 13,000 genes was analyzed (Table S7).

Using ORA, the pathway distribution was analyzed within the cytosol and cytomatrix, and the correspondence of pathways between the RNA-seq and Ribo-seq, at normal and drug-treated conditions. The Ribo-seq and RNA-seq data obtained by CMX over cytosol (CMX) revealed that transcriptome and translatome profiles differ in the CMX (Table S8). Data acquired of pp242 treated cells also showed transcriptome and translatome differences for the CMX (Table S9) and Cytosol (Table S10). The CMX translatome pathway differences are also present when comparing cells under pp242 treated and normal growth conditions (Table S11). Results indicate the flexibility of transcription and translation, which in turn may reflect the state of cells.

Additionally, the GOBP pathway differences were analyzed to determine corresponding pathways of transcription and translation between compartments (Tables S12–S14). The highest p-values for pathways identified by Ribo-seq for CMX corresponded to the lowest or undetected p-values of the cytosol (Table S12). The opposite result was obtained by comparing cytosolic pathways with the CMX (Table S13). The parallel Ribo-seq and RNA-seq analysis of the p values of the CMX and cytosolic pathways revealed that the lowest correlation occurs with the RNA-seq dataset (Table S14). The data also showed the overlapped pathways between the cytosol and the CMX. Overall results indicated that Ribo-seq analysis could be more reliable in showing real translation than RNA-seq, which prompted us to use ribosome footprints for the pathway analysis.

**Figure 4:**
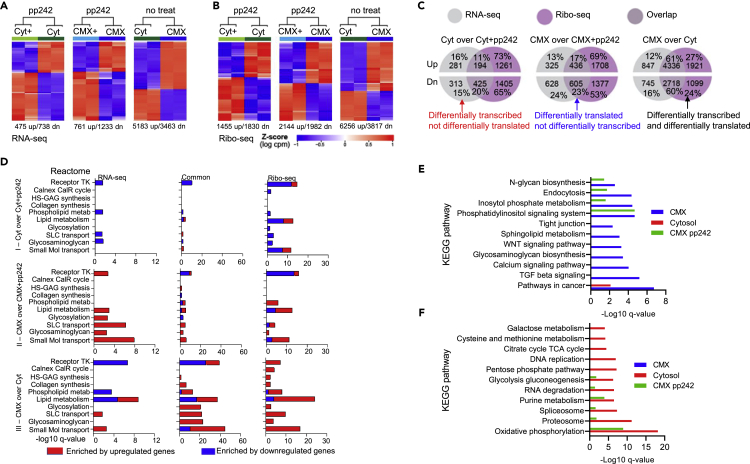
Analysis of transcription and translation in the cytomatrix and cytosol of HCT-15 cells (A) Heatmap of transcripts from RNA-seq. (B) Heatmap of footprints from Ribo-seq. (C) Venn diagrams of the overlaps and unique upregulated and downregulated transcript sets obtained from bulk RNA-seq and Ribosome footprint. (D) Reactome set visualization of the enriched upregulated and downregulated pathways. (E and F) KEGG analysis of enriched pathways identified through Ribo-seq.

Heatmaps for RNA-seq and Ribo-seq datasets, where the red color represents enriched by upregulated and the blue color is downregulated genes set, showed the distribution of transcripts in the cytosolic and CMX fractions that differed according to their respective *Z* score (Figures 4A and 4B). These hierarchical heatmaps also revealed differences between the pp242-treated and untreated cells for both transcriptome and translatome.

Venn diagrams represent the ratio of differentially transcribed (RNA-seq), translated (Ribo-seq), and overlapped transcript sets (Figure 4C). A low overlap rate was observed in the cytosol and CMX when the dataset was acquired for pp242 treated cells. The overlapped transcripts were higher in the CMX (CMX over Cyt) under normal growth conditions. These differences may reflect translational alteration between the cytosol and CMX compartments in normal and drug-treated cell states. The dynamics of the ribosome footprint and RNA-seq analyses indicated that some of the transcribed mRNAs (15%-16%) might not have been translated, which supports the notion that Ribo-seq analysis is more reliable in the identification of the enriched (ORA) pathways.

ORA of the select pathways of the Reactome and Hallmark collections are shown in Figures 4D and S5. RNA-seq, common, and Ribo-seq pathway dynamics were compared across the three groups of experimental conditions (I-III), which revealed the distribution of upregulated (red) and downregulated (blue) pathways. Experimental condition I shows the dynamics of cytosolic pathway distribution (Figures 4D-I and S5-I), which differed from the CMX dynamics (condition II). Experimental condition III, which identified CMX pathways under normal growth conditions, revealed upregulated and downregulated pathways that were specific to the CMX. The results revealed differences in upregulated and downregulated pathways, as well as transcription and translation, between the CMX and cytosol. Thus, ribosome footprint analysis exposed the highest upregulated pathway enrichment for the CMX (condition III, Ribo-seq).

Using ribosome footprint analysis, we assessed only the upregulated pathways to reveal translation differences between the cytosol and CMX (Figures 4E, 4F, S6A, and S6B). The CMX KEGG pathway analysis showed that some pathways enriched at the normal growth conditions differ from the CMX pathways of cells treated with the mTOR inhibitor (Figure 4E). Comparing the cytosolic and CMX pathways shows that pp242 treatment prompted pathway overlaps between the cytosol and CMX (Figure 4F). Differences were observed in the spectrum of pathways between the cytosol and CMX as well as pp242 treated and the normal growth conditions. Thus, the spectrum of pathways between the cytosol and CMX is altered depending on cell conditions.

Using the upregulated Reactome pathways, we analyzed differences between the cytosol and CMX (Figures S6A and S6B). The cytosolic pathways included translation, cell cycle, and respiratory pathways, but the overall PTM pathways overlapped (Figure S6A). The CMX pathways involved the ER-Golgi function that include transport, CNX/CalR cycle, lipid and steroid metabolism, and N-linked glycosylation, but the vesicle and membrane transport pathways overlapped (Figure S6B). The compartmental differences exposed functional dynamics, which supports previous selective western blotting data (Figures 1K and 2A). Transcribed and translated mRNAs, which have been used for RNA-seq and Ribo-seq analyses, could be associated with the CMX through mRNA binding complexes such as FUBP1/2, hnRNPK, and PTBP1/2 and other RNA binding proteins (Figure 1K and Table S1).

### Implications of the cytosolic and cytomatrix pathway differences

**Figure 5:**
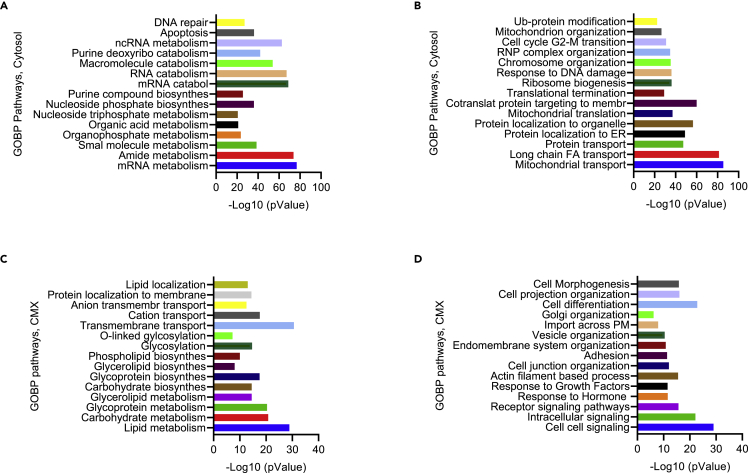
Upregulated pathways of the cytosol and cytomatrix of HCT-15 cells identified through Ribo-seq (A and B) Categorized cytosolic GOBP pathways. (C and D) Categorized cytomatrix GOBP pathways.

Using ribosome footprints, we compared the enriched GOBP pathways in the CMX and cytosol (Figures 5A-5D). The enrichment of RNA catabolism and the ubiquitin-proteasome pathways identified by the Ribo-seq analysis in the cytosol (Figures 5A and 5B) was supported by the mRNA and protein degradation markers detected by the western blot analysis within this compartment (Figures 1K, 2A, and 2B). Cytosolic enrichment of the upregulated pathways also included a small molecule, amide, organic acid, organophosphate, and nucleotide metabolism that can be categorized as a form of cellular nutrition. Specific transport, pathways involved in protein localization to organelles, translation, and organization of large protein complexes were enriched in the cytosol as well. The DNA repair, cell cycle transition, and apoptosis pathways were also enriched in the cytosol (Figures 5A and 5B), which corresponds with the KEGG and Reactome pathway Ribo-seq data analysis (Figures 4F and S6A).

Interestingly, the enrichment of the upregulated pathways within the cytomatrix differed from the cytosol (Figures 5C and 5D). Distribution of the carbohydrate, phospholipid, glycerolipid, and glycoprotein biosynthetic pathways within the CMX indicated immobilized biocatalysis. In living cells, bio-macromolecules are exposed to a densely crowded environment where a myriad of metabolic reactions that could exclude each other coincide. The immobilization of biosynthetic pathways provides the separation of chemical reactions and efficient biocatalysis. The upregulated pathways also included lipid, glycerolipid, carbohydrate, and glycoprotein metabolism. Consequently, the upregulated pathway enrichment also involved glycosylation and O-linked glycosylation, localization of proteins to membrane, and transmembrane transport (Figure 5C). Golgi organization, endomembrane system organization, actin filament-based processes, and cell junction organization pathways were also enriched in the CMX (Figure 5D). Response to hormones and Growth Factors, Receptor signaling pathways, intracellular and extracellular cell-to-cell signaling pathways were enriched as well. Apparently, these chains of the enriched pathways within the CMX form a logical system responsible for the cell phenotype. Accordingly, cell differentiation, cell projection organization and cell morphogenesis pathways are enriched in the cytomatrix (Figure 5D).

Although upregulated pathways in the CMX and cytosol appeared to be distinct, they nonetheless complement each other, which is essential for proper cell function. The examples of complementing pathways are the transporting systems and protein localization of the cytosol and CMX (Figures 5B and 5C).

The highest number of enriched pathways in the CMX were structural, metabolic, and signaling (Figure S7). Pathways related to protein glycosylation, transport, and translocation were also abundant in the CMX. The CMX pathways derived from the GOBP ribosome footprint are presented in Table S15. The upregulated and downregulated pathways that were enriched in the CMX and the cytosol are shown in Table S16. We observed that the relationship between the CMX and cytosol was inverse—downregulated pathways enriched in the CMX corresponded to upregulated pathways enriched in the cytosol.

A good correspondence was found between the ribosome footprints (Figure 5C) and protein expression, referring to the PTM, carbohydrate, and lipid metabolism pathways for example (Table 1). Ribo-seq pathway analysis, mass spectrometry proteome profiling, and western blotting experiments confirmed that the ER and Golgi matrices are integral parts of the CMX. Along with the cytoskeleton fibers, the ER and Golgi matrices are detergent and salt-resistant and form an elastic solid phase in contrast to mobile cytosol, which is detergent sensitive. Thus, the cytomatrix proteins and enzymes catalyze glycosylation, prenylation, or lipid transfer to proteins while remaining within the cytomatrix and forming protein cargo or secretory complexes. Cells would not be efficient if these enzymes were secreted with their cargo complexes to the extracellular space. Therefore, immobilized CMX enzymes are involved in lipid, carbohydrate, and glycolipid synthesis. These immobilized enzymes could also lead to glycosylation or lipidation posttranslational modifications, followed by the endomembrane system (ER) and Golgi organizations as well as transmembrane transport and ECM organization (Figure 5D). Thus, the cytomatrix integrates ER-Golgi-specific pathways with the cell surface receptors and signaling pathways.

## Discussion

In the mid-1970s, microscopy, immunocytochemistry, and cell fractionation methods revealed the cytoskeleton-associated ribosomes; however, the biological implication of this finding remained unclear due to the heterogeneous nature of the cytoskeletal fraction.^73^ Nevertheless, the considerable physiological significance of the mRNAs/polysomes association with the cytoskeleton has been stated. Still, it was also noted that the full implications of the molecular assembly must be awaited until detailed knowledge of the types of synthesized proteins is revealed. Keith R. Porter proposed the concept of a cytoplasmic structure termed the “microtrabecular lattice” (MTL), which was described as a three-dimensional meshwork.^74^^,^^75^^,^^76^ Since being proposed, however, the use of histological techniques has raised questions as to whether the MTL is a valid construct or artifact.^77^^,^^78^ The central principle underlying MTL was not just whether structure and order exist within the cytoplasmic matrix, but also whether the existing structure and order extend beyond the well-defined cytoskeletal fibers.^79^ Undoubtedly, the nano-compartments observed by HVEM^74^ could be the reason for the mRNA/polysome presence and distinct protein synthesis capacity of the CMX. Nano-compartmentalization concentrates the required components in a confined space, allowing an efficient formation of the end products.

Until now, there have been no significant attempts to chemically isolate the cytomatrix, which was predicted almost five decades ago.^76^ We used a two-step process to isolate the cytomatrix. First, the mild solvent separated the liquid phase (cytosol), and then the stringent solvent was applied to isolate the solid phase (cytomatrix) from the nucleus. The cytosol is a viscous fluid phase, consisting of water-soluble molecules and lipid-associated cargo, that is easily extracted with a nonionic detergent from the solid cytomatrix. Since the elastic solid phase is Li^+^ sensitive, a stringent buffer containing LiCl salt isolated the CMX. In choosing the name for the new elastic solid phase, we followed Dr. Porter’s CMX terminology.^74^

Coordination of the cytosol and CMX functions appear to play an essential role in fine-tuning cellular processes in healthy cells. High-throughput analysis and conventional methods revealed different responses from the cytosol and CMX during times of drug-induced protein deficiency. Of note, inhibiting mTOR evidently stimulated protein translation within CMX, suggesting the tenacity for protein synthesis. The inhibition of mTORC1 is required to initiate the autophagy process and proteolysis.^80^ The proteolysis that occurs in the cytosol, by providing amino acids, may stimulate protein synthesis in the CMX. The reprogrammed protein translation within the cytomatrix is likely a cell survival mechanism. Our data indicate that the CMX may potentially be essential in numerous metabolic reactions including energy generation, cell cycle, DNA replication and repair, and RNA processing depending on cell status and conditions. These functions could be a backup mechanism of the CMX in extreme conditions. Moreover, the compartmentalization of metabolic processes within the cytosol and CMX could support cancer cell survival and drug resistance as well as other pathological events. We propose that under normal growth conditions, the CMX is responsible for the cellular phenotype, whereas routine functions that support cell maintenance, such as nutrient processing and sustaining housekeeping reactions, occur in the cytosol.

To this end, the CMX appeared to be critical for performing specific tasks such as PTM of proteins, particularly glycosylation, which is the most abundant and diverse protein modification and is considered a special function of the ER and Golgi. The oligo/polysaccharide moiety could anchor (glue) the post-translationally modified proteins and protein complexes to form a matrix similar to the cell surface proteins embedded in a matrix of glycans.^81^ This concept of immobilized biocatalysis in the CMX is explained in Figure360 Video. We suggest that the cytomatrix filaments and motor proteins, such as actin, myosin, tubulin, and others, engages the cytosolic motion (a ripple) in reaction to extracellular receptor-ligand binding (Table 1, Figure 5D). Recent data have shown that the cytoplasmic Rho GTPases and actomyosin dynamics with the extracellular matrix can orchestrate the signaling output.^82^ The viscosity of the cytosol requires fluctuating cytoplasmic motion^7^ to transduce a cascade mechanism of immobilized biocatalysis and nuclear processes. Thus, the enzyme-substrate binding and immobilized biocatalysis could be regulated by cytosolic movement and dynamics of the specific receptor-ligand action.

The Golgi matrix is a detergent and salt-resistant complex.^18^ Our mild KCl-containing buffer would not dissociate Golgi, which has membrane clusters that are formed mainly by thick anastomosing tubular structures.^83^ The ER-to-Golgi interwoven tubular network is a stabilizing factor^84^ for the endoplasmic matrix organization. The ER proteinaceous carcass revealed by the cryo-ET^45^ also appeared to be salt and detergent-resistant. Thus, Golgi and ER networks are integral parts of the cytoplasmic matrix along with cytoskeleton fibers. The Golgi and ER matrices are detergent and salt-resistant. However, the water and lipid-soluble mobile secretory materials, or cargo, of the Golgi and ER extracted to the cytosol are detergent-sensitive. The distribution of Golgi and ER proteins in the CMX fraction and the role of detected proteins in glycosylation and transportation suggests a unique function of the CMX in the biosynthetic-secretory pathway, that affirms the ER-Golgi complex as a segment of the elastic solid phase. In addition, the role of ER-Golgi as a segment of CMX in transport and cell-surface events (cell adhesion, ECM formation) implies a connection of the intracellular matrix with the extracellular matrix. Thus, a matrix system segregating biocatalysis performs efficient reactions and signaling.

The coinciding metabolic reactions that could exclude each other may interrupt biochemical reactions in a confined space. However, the immobilization of biosynthetic pathways, via the CMX, provides separated chemical reactions and efficient biocatalysis, that overcomes spatial impediments. Efficient biocatalysis, achieved by segregating and immobilizing the metabolome enzymes through the lipid and carbohydrate PTM, may form corresponding organelles that perform specific reactions and processes within the cytoplasm. Thus, the presence or absence of organelles in tissue-specific cells and reorganization of organelles after cell division depends on biosynthetic pathway immobilization. The dynamics of the cytoplasmic organelles, apart from DNA containing mitochondria, may depend on the organization of the CMX. Consequently, the cytomatrix organizes Golgi-ER pathways into a singular integrated system along with the receptors and signaling pathways. As such, we define the CMX as the flexible solid state of the cytoplasm, and a separate but interdependent entity from the viscous fluid (liquid) state of the cytosol. Immobilized biocatalysis integrates the intracellular and extracellular matrices and receptors with the nuclear processes, thus removing the spatial hindrances for biochemical processes, which elucidates cellular mechanics and cytoplasm organization.

### Limitations of the study

The high-throughput experiments (Ribo-seq, RNA-seq, and proteomics) were only performed in HCT-15 cells. Comparative studies such as between normal and cancer cells are needed to expand the results obtained using this new approach.

## STAR★Methods

### Key resources table

**Table:** 

REAGENT or RESOURCE	SOURCE	IDENTIFIER
**Antibodies**		

pan-Keratin	Cell Signaling Technology	Cat# 4545; RRID:AB_490860
Emerin	Cell Signaling Technology	Cat# 5430; RRID:AB_10691714
ERp57	Cell Signaling Technology	Cat# 2881; RRID:AB_2160840
B23-NPM	Cell Signaling Technology	Cat# 3542; RRID:AB_2155178
Nup98	Cell Signaling Technology	Cat# 2598; RRID:AB_2267700
β-actin	Cell Signaling Technology	Cat# 3700; RRID:AB_2242334
eEF2k	Cell Signaling Technology	Cat# 3691; RRID:AB_2097313
eIF4E	Cell Signaling Technology	Cat# 9742; RRID:AB_823488
4E-BP1	Cell Signaling Technology	Cat# 9644; RRID:AB_2097841
eIF4B	Cell Signaling Technology	Cat# 3591; RRID:AB_2097522
phospho-T37 4E-BP1	Cell Signaling Technology	Cat# 2855; RRID:AB_560835
phospho-T70 4E-BP1	Cell Signaling Technology	Cat# 9455; RRID:AB_330949
non-phospho-T46 4E-BP1	Cell Signaling Technology	Cat# 4923; RRID:AB_659944
phospho-S65 4E-BP1	Cell Signaling Technology	Cat# 9451; RRID:AB_330947
mTOR	Cell Signaling Technology	Cat# 2983; RRID:AB_2105622 #4517; RRID:AB_1904056
p70S6K1	Cell Signaling Technology	Cat# 9234; RRID:AB_2269803
Ero-1Lα	Cell Signaling Technology	Cat# 3264; RRID:AB_823684
PDI	Cell Signaling Technology	Cat# 3501; RRID:AB_2156433
DCP1B	Cell Signaling Technology	Cat# 13233; RRID:AB_2798157
DDX6	Cell Signaling Technology	Cat# 9407; RRID:AB_10556959
Tubulin	Cell Signaling Technology	Cat# 2128; RRID:AB_823664
CalR	Cell Signaling Technology	Cat# 2891; RRID:AB_2275208
PKCζ	Cell Signaling Technology	Cat# 9368; RRID:AB_10693777
PKC δ	Cell Signaling Technology	Cat# 2058; RRID:AB_10694655
BiP	Cell Signaling Technology	Cat# 3177; RRID:AB_2119845
LC3B	Cell Signaling Technology	Cat# 3868; RRID:AB_2137707
EEA1	Cell Signaling Technology	Cat# 3288; RRID:AB_2096811
AIF	Cell Signaling Technology	Cat# 5318; RRID:AB_10634755
Rab5	Cell Signaling Technology	Cat# 3547; RRID:AB_2300649
RCAS1	Cell Signaling Technology	Cat# 12290; RRID:AB_2736985
Calnexin (CNX)	Cell Signaling Technology	Cat# 2679; RRID:AB_2228381
PTBP1/2	Cell Signaling Technology	Cat# 8776 The product is discontinued
hnRNPK	Cell Signaling Technology	Cat# 9081; RRID:AB_11178946
FUBP/ KHSRP (E2E2U)	Cell Signaling Technology	Cat# 13398; RRID:AB_2798208
IRE1α	Cell Signaling Technology	Cat# 3294; RRID:AB_823545
rpS6	Cell Signaling Technology	Cat# 2217; RRID:AB_331355
rpL7a	Cell Signaling Technology	Cat# 2415; RRID:AB_2182059
SUN2	Abcam	Cat# ab124916; RRID:AB_10972497
Nesprin 1/Syne-1	Abcam	Cat# ab5250; RRID:AB_2200724
Exportin 5	Abcam	Cat# ab57491; RRID:AB_941499
SUN2	Sigma-Aldrich	Cat# MABT880
GRP78/HSPA5/BiP	Novus Biol	Cat# 06274; RRID:AB_1555284
eIF4E3	Proteintech	Cat# 17282-1-AP; RRID:AB_2262162

**Bacterial and virus strains**		

ShRNA_eIF4E3 lentiviral particles	Santa Cruz Biotechnology	Cat# sc-78455-V
Mammalian vector pCMV6-AC-GFP (Human)	Origene	Cat# PS100010

**Chemicals, peptides, and recombinant proteins**		

pp242	Tocris Bioscience	Cat# 4257/5
Kinesin inhibitor K858	Tocris Bioscience	Cat# 4960/1
Dynarrestin	Tocris Bioscience	Cat# 6526/5
Torin 1	Tocris Bioscience	Cat# 4247/10
PF4708671	Tocris Bioscience	Cat# 4032/5
Bio	Tocris Bioscience	Cat# 3194/10
Rotenone	Tocris Bioscience	Cat# 3616/50
MG132	Tocris Bioscience	Cat# 1748/5
Geldanamycin	Tocris Bioscience	Cat# 1368/1
T4 Polynucleotide Kinase	New England Biolabs	Cat# M0201S
T4 PK Reaction Buffer	New England Biolabs	Cat# B0201S
DMEM	Thermo Fisher Scientific	Cat# A4192101
RPMI 1640	Thermo Fisher Scientific	Cat# 61870143
FBS	Thermo Fisher Scientific	Cat# A4766
Penicillin/Streptomycin	Sigma	Cat# P4333
HEPES solution	Sigma	Cat# H0887
NaCl	Sigma	Cat# S5150
Glycerol	VWR	Cat# 24388-295
NP-40/IPEGAL	EMD Millipore	Cat# 492016
Triton X-100	Sigma	Cat# T8787
Tris-HCl pH 7.5 1M	Thermo Fisher Scientific	Cat# 15567027
RNAsin RNase inhibitor	Promega	Cat# N251B
Sodium deoxycholate	Ameresco	Cat# 0613
KCl	Thermo Fisher Scientific	Cat# AM9640G
MgCl_2_	Thermo Fisher Scientific	Cat# AM9530G
LiCl	Invitrogen	Cat# L7026
DTT	OmniPur	Cat# 3860
Cycloheximide	Ameresco	Cat# 94271-5G
RNase-free water	GE Healthcare	Cat# 0226
siRNA buffer	Dharmacon™	Cat# B-002000-UB-100
Protease Inhibitors	Roche	Cat# 11393100
qScript cDNA Super-Mix	QuantaBio	Cat# 10142-784
Formaldehyde	Sigma	Cat# F9037
MNase	Sigma-Aldrich	Cat# N3755
Guanidine thiocyanate	Sigma	Cat# 50983
Trizol	Ambion	Cat# 10296-010
GlycoBlue	ThermoFisher Scientific	Cat# AM9515
Polynucleotide kinase	BioLabs	Cat# M0201S
PBS	Gibco	Cat# 14200-075
Tween 20	Sigma-Aldrich	Cat# H06J02
Puromycin	GIBCO	Cat# J67236.8EQ
BSA	Sigma-Aldrich	Cat# A9647

**Other**		

Compound	Target	Concentration
pp242	mTOR	500 nM
K858	Kinesin	1–10 µM
Dynarrestin	Dynein	5–25 µM
Torin 1	mTOR	250 nM
PF4708671	RSK	5 µM
Bio	GSK-3	1 µM
Rotenone	Mitochondrial electron transport	1 µM
MG132	26S proteasome	1 µM
Geldanamycin	Hsp90	1 µM

**Critical commercial assays**		

RNeasy Mini Kit	QIAGEN	Cat# B-002000-UB-100
Stellaris RNA FISH	Biosearch technologies	Cat# VSMF-20680-5
QIAseq miRNA Library Prep Kit	QIAGEN	cat #331502
MTT Cell Growth Assay Kit	Sigma-Aldrich	Cat# CT02

**Deposited data**		

mRNA and Ribo-seq data	NCBI GEO	GSE199535
The mass spectrometry proteomics data	ProteomeXchange Consortium	PRIDE: PXD031043

**Experimental models: Cell lines**		

Colorectal carcinoma HCT-15	ATCC	CCL-225
Cervical adenocarcinoma HeLa	ATCC	CCL-2
Gastric adenocarcinoma AGS	ATCC	CRL-1739
Hepatocellular carcinoma Hep G2	ATCC	HB-8065

**Experimental models: Organisms/strains**		

HCT-15, GFP- rpL7a expression	Origene	Cat# PS100010
HeLa, siRNA gene silencing, SUN2	Dharmacon™	Cat# 25777
HCT-15, Transduction of shRNA eIF4E3 lentiviral particles	Santa Cruz Biotechnology	Cat# sc-78455-V

**Oligonucleotides**		

Human SUN2 siRNA	Dharmacon™	Cat# 25777
ShRNA_eIF4E3 lentiviral particles	Santa Cruz Biotechnology	Cat# sc-78455-V

**Software and algorithms**		

Proteome Discoverer 2.1 software	Thermo Scientific	https://www.thermofisher.com/us/en/home/industrial/mass-spectrometry/liquid-chromatography-mass-spectrometry-lc-ms/lc-ms-software/multi-omics-data-analysis/proteome-discoverer-software.html
Mascot 2.4	Matrix Science	https://www.matrixscience.com/
gpGrouper algorithm	Saltzman et al. (2018, MCP)^85^	PMID: 30093420
Gene Set Enrichment Analysis (GSEA)	Subramanian et al. (2005, PNAS)^86^	https://www.gsea-msigdb.org/gsea/index.jsp
FastQC	Andrews et al. (2010)^87^	https://www.bioinformatics.babraham.ac.uk/projects/fastqc/
Trimmomatic (v0.38)	Bolger et al., 2014^88^	Bioinformatics 30, 2114-2120
Bowtie aligner (v1.3.0)	Langmead et al., 2009^89^	Genome Biology 10, R25.
The STAR aligner (v2.7.6a)	Dobin et al., 2013^90^	Bioinformatics 29, 15-21.
R package edgeR	Robinson et al., 2010^91^	Bioinformatics 26, 139-140
RUV normalization	Risso et al., 2014^92^	Nature biotechnology 32, 896-902
MSigDB	Liberzon et al., 2011^93^	Bioinformatics 27, 1739-1740
MATLAB	Mathworks	https://www.mathworks.com/products/matlab.html
Image Studio Lite	LI-COR	https://www.licor.com/bio/image-studio-lite/
GraphPad Prism 9.4.1	GraphPad	https://www.graphpad.com/scientific-software/prism/
Adobe Illustrator CC 2022	Adobe	https://www.adobe.com/products/illustrator.html
NCBI GEO RNA-seq data	GEO accession GSE199535	https://www.ncbi.nlm.nih.gov/gds
ProteomeXchange Proteomic data	Dataset Identifier PXD 031043	http://proteomecentral.proteomexchange.org/cgi/GetDataset

### Resource availability

#### Lead contact

Further information and requests for resources and reagents should be directed to and will be fulfilled by the lead contact, Tattym Shaiken (tattyms@bcm.edu).

#### Materials availability

This study did not generate new unique reagents.

### Experimental model and subject details

#### Cell lines and cell culture

Colorectal carcinoma HCT-15 (CCL-225, RPM-1640), cervical adenocarcinoma HeLa (CCL-2, DMEM), gastric adenocarcinoma AGS (CRL-1739, DMEM), and hepatocellular carcinoma HepG2 (HB-8065, DMEM) cells were grown in the incubator under humidified conditions at 37°C under 5% CO_2_ in Dulbecco’s Modified Eagle Medium or RPMI-1640 supplemented with 10% (v/v) fetal bovine serum (FBS) and 1% (v/v) penicillin and streptomycin. The cells were treated with the indicated concentration of the compounds for 24 h or as indicated in the experiment. Cells were grown to 90-95% confluency.

#### Generation of fluorescently labeled cell line

HCT-15 cells were grown on 35 mm glass bottom dishes and transfected with the GFP-L7a vector pCMV6-AC-GFP at a concentration of 1 μg/dish. At 4 h post-transfection, HCT-15 cells were treated with one of the following compounds for 24 h: 500 nM of pp242, 1 μM Rotenone, 250 nM Torin 1, 1 μM Bio, 1 μM Geldanamycin, 5 μM PF4708671, and 1 μM MG132. To detect the subcellular localization of GFP-tagged proteins, confocal microscopy (Zeiss LSM 780) images of live cells were taken after 24 h of exposure to the tested compound.

#### siRNA gene silencing

We designed ON-TARGET plus non-targeting pool siRNA (control) and ON-TARGET plus Human SUN2 siRNA (25,777) in a SMARTpool format. HeLa cells grown at a density of 1 × 10^5^ cells in four chamber slides were transfected with siRNAs using DharmaFECT transfection reagent according to the manufacturer’s recommended method. The final concentration of siRNA was 25 nM. After transfection, cells were incubated at 37°C for 72 h before further analysis. The efficiency of the siRNAs was determined by immunofluorescence.

#### Transduction of shRNA eIF4E3 lentiviral particles

We validated the specificity of an anti-eIF4E3 antibody by shRNA knock-down. Sh_eIF4E3 lentiviral particles (Santa Cruz Biotechnology Inc., Dallas TX) were used to knock-down the expression of eIF4E3 in HCT-15 cells according to manufacturer’s instruction. On day 2, HCT-15 cells were transduced with 15 μL of sh_eIF4E3 lentiviral particles. We selected stable clones expressing eIF4E3 shRNA by adding 10 μg/mL Puromycin. On day 5, the transduction efficiency was estimated by detecting cells generating green fluorescence protein under fluorescence microscope.

### Method details

#### Buffer ionic composition calculation

Standard approaches to separating the aqueous cytoplasm^94^ have not been sufficient for isolating the intracellular CMX. The sequential fractionation of the cytoplasm to obtain cytosol and membrane-bound proteins^95^ has likewise proven insufficient for CMX separation. A hypotonic solution is often used to isolate the nucleus. The PM of mammalian cells easily ruptures with a low salt hypotonic solution.^96^ The osmotic shock caused by a change in the solute concentration (hypotonic or hypertonic) can rupture PM and organelles. Rupturing the PM and other organelles allows the cytoplasmic contents of the cell to leak out while leaving the nucleus intact.^97^ In hypertonic conditions up to 0.6M NaCl, solubilization of nucleosomes was achieved.^98^ High salt disrupts charged-based protein-DNA, and protein-protein interactions and chromatin-associated proteins become more soluble.^99^ To isolate the cytosol and CMX at physiological osmotic conditions, we developed an equation to calculate the concentration of ions needed to replace sodium ions in a buffer based on the relative ionic radii for the Hofmeister series of monovalent cations (Figure S1B). Physiological or isotonic saline is a solution of 154 mM (0.90% w/v) NaCl, 308 mOsm/L. The relative radii used for the Hofmeister series monovalent cations were as follows: Li = 90 pm., Na = 116 pm., and K = 152 pm.^28^ The relative ionic radius is a solvation shell of ions in solution that surround proteins or protein complexes in the cell. The ionic ratio of protein solutions depends on the relative radius of ions. The concentration of replacing cations was calculated using the equation Cr = 18/Ri, where Cr is the replacing concentration and Ri is the ionic radius. Because the dominant positive cation within the cell is potassium,^100^ we used potassium chloride to mimic the natural ionic environment of the cell. Using this equation, we found that 120 mM KCl and 200 mM LiCl corresponded to 154 mM NaCl. These concentrations of ions were used sequentially in the isolation of the cytosol and the CMX. We used a minimal solvent volume for cell lysis to induce less damage to the CMX and to maintain the ionic balance.

#### Cell fractionation

We treated HCT-15 cells with 500 nM of pp242 every 6 h for 24 h unless otherwise stated. Control cells were grown under normal growth conditions. Experiments were performed in duplicate using 10 cm dishes. At least three 10 cm dishes were used for the CMX isolation experiment.

1. The growth media from control untreated HCT-15 cells at an exponential growth phase and pp242-treated cells at cell growth arrest were aspirated and 10 cm dishes with cells were placed on ice in tilted position. All the experiments were performed on ice or in cold room that slows down metabolic processes (Figure S1C).
2. Cells were washed with ice-cold PBS twice allowing the PBS to flow down from tilted dishes and be aspirated.
3. Cells were lysed using cell scrapers in an appropriate volume Buffer A (0.1 mL Buffer A per 10 cm dish or 0.3 mL Buffer A for 30 cm cell culture dish) and placed into Eppendorf tubes. At this volume of the Buffer A, the lysate volume would equal 0.2 mL (10 cm dish) and 0.6 mL (30 cm dish), respectively. Thus, the cell lysate would be diluted only 2-fold.
4. Buffer A composition is 40 mM HEPES pH 7.4, 120 mM KCl, 0.5% Glycerol, and 0.5% NP-40 with protease inhibitors. The 120 mM KCl mimics the intracellular ionic composition that prevents the disintegration of the cytomatrix. The nonionic detergent NP-40 solubilizes the viscous fluid content of the cytoplasm, extracting water soluble biopolymers, neutral lipids, and phospholipids of the PM, ER, mitochondria, Golgi, and other lipid containing droplets, and cargo molecules from the cytomatrix.
5. Scraped cell lysates rotated for 30 min at 4°C. Mixing the collected cells lysates by rotation with Buffer A for 30 min provides time for micelles to form, which is a slow process at cold conditions. The micelles envelope lipids and proteins immersed in lipid bilayers.
6. Lysates were centrifuged at 500 × g for 5 min at 4°C, and the supernatants were collected as the cytosol. The cytosol represents colloid suspension of the micelles. Low speed centrifugation prevents breaking of the micelles and reabsorption to the pelleted CMX.
7. The pellets were gently washed with two volumes of washing Buffer, which is 40 mM HEPES pH 7.4, 120 mM KCl, 0.5% Glycerol. The pellet was gently resuspended tapping the tube. The wash can be discarded or added to the cytosol fraction. Washing the pellet without detergent enables Buffer A to be replaced with the next buffer, Buffer B.
8. The pellet was resuspended by gently pipetting it seven times in Buffer B. Buffer B has a composition of 10 mM Tris-HCl pH 7.5, 1.5 mM KCl, 0.5% Triton X-100, 0.5% sodium deoxycholate, 2.5 mM MgCl_2_, 0.2 M LiCl, and protease inhibitors. The 0.2M LiCl was added to the Buffer B just before the experiment. Replacing potassium ions with lithium condenses the nuclear content, breaks LINC complex and dissolves the cytomatrix that allows for separation of the core nuclear content from the elastic solid elements of the cytoplasm.
9. The resuspension of the pellet by cold rotation continued for 45 min to complete solubilization.
10. The cytomatrix was isolated by centrifuging at 2000 × g for 5 min. The supernatants were collected as the CMX.
11. The pellets were washed with two volumes of Buffer B and combined to CMX or discarded.
12. The nuclear pellet was dissolved in 8M urea.
13. Cytosol, CMX, and nuclear fractions were clarified at 10,000 × g for 10 min.
14. The protein concentration was determined by Lowry. For RNA extraction and polysome profiling, all buffers were prepared in RNase-free water with the same buffer compositions and the addition of 500 U/ml RNasin. All procedures were performed at 4°C.

#### Polysome profiling

For the polysome profiling, 2 mM DTT and 100 μg/mL Cycloheximide (CHX) was added to the PBS. All buffers were prepared in RNase-free water. The CHX (100 μg/mL) was added to 75% confluent HCT-15 cells (control cells and cells treated with pp242) for 30 min before cell lysis. The cytosol and CMX were obtained as described. Approximately 2 mg of proteins were overlaid onto 10–50% sucrose gradients and centrifuged at 100,000 × g for 3 h. The polysome profile was monitored at 254 nm (Brandel, BR-188-176; Gradient Fractionation System).

#### Incorporation of labeled amino acids into proteins

HCT-15 cells were treated with 500 nM of pp242 for 24 h. Incorporation of radiolabeled amino acids into proteins was performed as described previously.^101^ Briefly, pp242 treated and non-treated HCT-15 cells were incubated with L-[^3^H] phenylalanine (5 μCi/mL) for 1 h. After incubation, the medium was removed and the cells were washed twice with ice-cold PBS. The cells were fractionated, and the protein concentration was determined by the Lowry method. Approximately 100 μg of protein from each fraction were blotted onto Whatman 3 MM paper filters and allowed to dry. The dried filters were immerged in 50 mL of 5% (w/v) TCA and boiled for 2 min. The procedure was repeated once. The filters were rinsed briefly in 100% ethanol and dried. Finally, the radioactive signal (in cpm) was counted on a Beckman LS 6500 LSC.

#### RNA -seq

For Next-Generation Sequencing (RNA-seq), HCT-15 cells were treated with 500 nM pp242 for 6 h, with an additional treatment at 3 h. Control cells were grown under normal growth conditions. Total RNA was isolated from the cytosol and CMX using a RNeasy Mini Kit (QIAGEN, Germantown, MD) according to the manufacturer’s instructions. Total RNA samples were normalized to 200 ng each and libraries were generated using the NEB Ultra II Directional RNA Library Prep kit according to the kit specifications. All samples were pooled equimolarly and sequenced on an Illumina NovaSeq 6000 flow cell with paired-end reads (PE150). An average of 51 million read pairs per sample were sequenced.

#### Ribo-seq

The cytosol and CMX from control and treated cells were obtained as previously described and digested with micrococcal nuclease (MNase, Sigma-Aldrich, St Louis, MO) at 37°C for 30 min to obtain ribosome footprints. The reaction was stopped by the addition of 1.5 volumes of 4 M guanidine thiocyanate. Footprints were extracted by adding TRIzol and isolated by precipitating with GlycoBlue (ThermoFisher Scientific, Houston, TX) overnight.^102^ The footprints were treated with polynucleotide kinase (ENK) at 37°C for 30 min to reverse the phosphate position. We separated 17–37 nt long footprints from the gel under UV light. We froze gel slices at −80°C and then crushed them to extract RNA.

#### Library Preparation

We used 10 ng of size-selected ribosome-protected RNA (17–37 nt) as the starting material for the QIAseq miRNA Library Prep Kit (cat #331502). RNA fragments were ligated to adapters at the 3′ and 5′ ends, reverse transcribed, and amplified. The resulting libraries were size selected for 185–191 bp fragments. The libraries were quantitated by qPCR using the Applied Biosystems ViiA7 qPCR instrument and a KAPA Library Quant Kit (p/n KK4824). All samples were pooled equimolarly and sequenced on a NextSeq 500 High Output v2.5 flowcell (Illumina p/n 20024906) using the Illumina NextSeq 500 sequencing instrument with a single-read configuration (75 bp). An average of 42 million reads per sample was sequenced. FastQ file generation was executed using Illumina’s cloud-based informatics platform, BaseSpace Sequencing Hub.

#### Bulk RNA-seq and Ribo-seq analysis

Two biological replicates of ribosomal footprint and mRNA libraries were generated and subjected to deep sequencing to obtain the Ribo-seq and RNA-seq datasets. The sequence files generated by the RNA-seq and Ribo-seq experiments were trimmed using Trimmomatic (v0.38),^88^ and sequence quality was assessed using FastQC (v0.11.8).^87^ Next, we used the bowtie aligner (v1.3.0) to remove rRNA sequences from the Ribo-seq experiment.^89^ The rRNA sequences used in the rRNA depletion step were downloaded from Ensembl BioMart. The STAR aligner (v2.7.6a) was then used to align and quantify gene expression for both the Ribo-seq and the RNA-seq sequence,^90^ using the human genome build GRCh38 p13 v35. Differential gene expression of protein coding genes was evaluated using the R package edgeR,^91^ with upper quartile and RUV (remove unwanted variation) normalization.^92^ Significance was achieved for a fold change exceeding 1.5× and an FDR-adjusted p value < 0.05.

#### Over-representation analysis (ORA)

ORA was performed to detect enriched gene sets corresponding to pathways and biological processes based on differential gene expression. Using the Hallmark, KEGG, Reactome, and Gene Ontology Biological Process compendia (v7.3) and the Molecular Signature Database methodology (MSigDB),^93^ we assessed enrichment with a hypergeometric test. Significance was achieved at an FDR-adjusted p value <0.05.

#### LC-MS/MS analysis

Two biological replicates were used for LC-MS/MS analysis. The cytosol and CMX fractions were concentrated and digested on an S-Trap column (Protifi, NY) per the manufacturer’s protocol. Offline high pH STAGE peptide fractionation (15 fractions combined to 5 peptide pools) for 50 μg peptides was carried out as previously described (PMID: 30093420). LC-MS/MS analysis was carried out using a nano-LC 1200 system (Thermo Fisher Scientific, San Jose, CA) coupled to Orbitrap Lumos ETD mass spectrometer (Thermo Fisher Scientific, San Jose, CA). Peptide (1 μg) was loaded onto a 2 cm × 100 μm internal diameter (ID) pre-column switched in-line with an in-house 20 cm × 75 μm ID column (Reprosil-Pur Basic C18, 1.9 μm, Dr.Maisch GmbH, Germany) equilibrated in 0.1% formic acid/water. The column temperature was maintained at 60°C. The peptides were eluted using a 110 min gradient of 2–30% acetonitrile/0.1% formic acid at a flow rate of 200 nL/min. The mass spectrometer was operated in the data-dependent acquisition mode using the top30 method. MS1 was acquired in the Orbitrap (120,000 resolution, 350–1400 *m/z*) followed by MS2 in the IonTrap (HCD 32%, AGC 2E4, 30 ms ion injection) with a 15 s dynamic exclusion time.

The RAW files were processed using the Proteome Discoverer 2.1 software (Thermo Scientific) using Mascot 2.4 (Matrix Science) with percolator against human NCBI refseq database updated on 2020_0324. The precursor ion tolerance and product ion tolerance were set to 20ppm and 0.5Da, respectively. The enzyme was set to Trypsin/P with maximum missed cleavage of 2 the dynamic modifications of oxidation, carbamidomethyl on cysteine, and protein N-terminal acetylation were allowed. The peptides identified from the Mascot result file were validated with 5% false discovery rate (FDR). The gene product inference and iBAQ-based quantification was carried out using the gpGrouper algorithm.^85^ The median normalized iBAQ values were used for data analysis. The differentially expressed proteins were calculated using the moderated t-test to calculate p values and log2 fold changes in the R package limma. The FDR corrected p value was calculated using the Benjamini-Hochberg procedure. Gene Set Enrichment Analysis (GSEA) (PMID: 16199517) was performed using the canonical pathway gene sets derived from the KEGG and Reactome pathway databases.^86^

#### Immunoblotting

Total protein in the cell fractions was measured, equalized by concentration, and boiled at 95°C for 5 min in sample buffer. The samples were then resolved using a 4–15% Mini-PROTEAN TGX Precast Gel (Bio-Rad) at 100V for 100 min. The resolved proteins were transferred onto Immobilon PVDF membranes (Thermo Fisher Scientific) at 200 mA for 60 min at 4°C. The membranes were blocked in Odyssey Blocking Buffer (Li-Cor) and probed with the respective primary antibodies at 1:1,000 dilution overnight at 4°C. The membranes were washed three times for 5 min with Tris-buffered saline with 0.05% Tween 20 (Sigma-Aldrich) and incubated with the secondary (Santa Cruz Biotech) Horseradish Peroxidase (HRP)-antibodies (1:20,000) for 1 h at room temperature on the rocker. The blots were detected by LiCor Odyssey scanner.

#### Immunolabeling

Cells were immunolabeled and visualized by deconvolution (GE Healthcare) and confocal (Nikon) microscopy. Per chamber, 20,000 cells were seeded and cultured for 24 h. Cultured cells were fixed with 4% PFA, permeabilized with 0.2% (v/v) Triton X-100 in PBS for 10 min at RT, and blocked with horse or goat serum for whole cell imaging. Cell fractions (including nuclei with or without the cytosol) were deposited onto microscope slides using a cytospin at 4°C, fixed with 4% PFA, and blocked with horse serum. The slides were processed using a standard immunostaining protocol. The cells and cell fractions were incubated with the appropriate primary antibodies overnight at 4°C, washed in PBS, and incubated with Alexa 488 and/or Alexa 594-conjugated secondary antibodies for 1 h at 4°C.

#### Fluorescent *in situ* hybridization (FISH)

To detect ribosomal S6 (RPS6) mRNA, we performed single-molecule FISH using Stellaris RNA FISH probes conjugated to CAL Fluor Red 610 targeting Human RPS6 (VSMF-20680-5), Biosearch technologies, Petaluma, CA). Control and pp242 treated HCT-15 cells on cover glasses were fixed in PBS containing 3.7% (v/v) formaldehyde and permeabilized with 70% (v/v) ethanol for 1 h at 4°C. The cells were then washed with 10% formamide buffer (Stellaris Wash Buffer A) before hybridizing with the probe set in Stellaris Hybridization Buffer (SMF-HB1-10). The hybridizations were performed overnight in a moist chamber at 37°C. The following day, the cells on cover glasses were transferred to Stellaris Wash buffer A (SMF-WA1-60) and incubated for 30 min. For nuclear staining, we incubated cells with DAPI (5 ng/ml) for 30 min in the dark in Wash buffer A, washed with Stellaris Wash buffer B (SMF-WB1-20) for 5 min and mounted. Images were obtained using a deconvolution microscope (GE Healthcare, Olympus IX71).

#### Transmission electron microscopy (TEM)

HCT-15 cells were fractionated as described above. Cellular and subcellular fractions were fixed in 3% glutaraldehyde (PBS, pH 7.3) for two days, washed with sodium phosphate buffer (pH 7.3), post-fixed with 1% osmium tetroxide for 1 h, and dehydrated through a series of graded alcohol baths. Standard TEM preparation techniques were used to obtain sample blocks that were cut in ultra-thin sections (80 nm) for mounting onto 100 mesh copper grids. The grids were stained with 2% alcoholic uranyl acetate and Reynold’s lead citrate. The grids were examined with a Zeiss CEM 902 transmission electron microscope, and images were captured using an AMT602 digital camera.

#### High throughput nucleolar hypertrophy assessment

To analyze nucleolar morphology of HCT-15 cells, we performed high speed imaging with optimum focusing capabilities. We seeded 20,000 cells in a 384 well-plate (Greiner) and the next day, we treated cells with 500 nM of pp242, 1 μM of MG132, and 1 μM of Rotenone for 24 h. Cells were fixed with 4% PFA, permeabilized by 1% Triton X-100 for 1 h, blocked with goat serum, incubated with anti-L7a antibody overnight, and detected using Alexa 488-conjugated secondary antibody. Nuclei were stained with DAPI. At least 200 cell nuclei were analyzed with four repeats. Cells were imaged on an IC200 high-throughput image cytometer (VALA Sciences) using a plan APO 40× objective. A single plane was acquired for the DAPI channel, and a z stack series (10 μm, 1 μm increment) was acquired for the GFP channel. Image analysis was performed using a custom-made algorithm developed in MATLAB. We analyzed two fields-of-view (FOV) per well (average of 46 cells per FOV). For each FOV, DAPI-labelled nuclei were segmented using a watershed transform strategy. For nucleolus segmentation, the best focused GFP-labelled nucleoli plane was selected, and objects were identified by first performing morphological top-hat filtering, followed by computing the regional maxima of the H-maxima transform. The mean number of nucleoli per nucleus, average size of nucleoli, and mean fluorescence intensity of nucleoli were then calculated.

#### RT-PCR

For the RT-PCR, total RNA was isolated from cytosol and CMX of HCT-15 cells using RNeasy Mini Kit (QIAGEN). qScript cDNA Super-Mix (QuantaBio) was used for cDNA synthesis. Beta-actin and 18S rRNA primers (Invitrogen) were used for PCR reaction.

### Quantification and statistical analysis

Data are presented as the mean ± SD. One-way ANOVA was used to detect significant differences between the means of more than two independent groups using Prism 6 (GraphPad). We independently performed t-tests to confirm statistically significant differences between two groups. A p value of <0.05 was considered as significant.

## Data Availability

This paper does not report original code. Data reported in this paper will be shared by the lead contact upon request. The RNA sequencing data was uploaded to NCBI GEO and is publicly available as of the date of publication. Accession numbers are listed in the key resources table. The mass spectrometry proteomics data have been deposited to the ProteomeXchange Consortium via the PRIDE partner repository and is publicly available as of the date of publication. The dataset identifier is listed in the key resources table. Any additional information required to reanalyze the data reported in this paper is available from the lead contact upon request.

## Supplemental information

Document S1. Figures S1-S7 and Tables S8-S14

Table S1. Mass-spec proteome category, related to Figure 3

Table S2. Mass-spec iBAQ, related to Figure 3

Table S3. Hallmark pathway, related to Figure 4

Table S4. KEGG pathway, related to Figure 4

Table S5. Reactome pathway, related to Figure 4

Table S6. GOBP analysis, related to Figure 4

Table S7. Genes and q values, related to Figure 4

Table S15. Cytomatrix activity, related to Figure 5

Table S16. GOBP up and down-regulated pathways of CMX and cytosol, related to Figure 5

Figure360. An author presentation of graphical abstract
