# Supplementary material for: Transcriptome, proteome, and protein synthesis within the intracellular cytomatrix
Source: iScience. 2023 Jan 13;26(2):105965. doi: 10.1016/j.isci.2023.105965 (PMC9941065; doi:10.1016/j.isci.2023.105965)
Supplement: Document S1. Figures S1-S7 and Tables S8-S14 [file mmc1.pdf]

iScience, Volume *26*

Supplemental information

Transcriptome, proteome, and protein synthesis within the intracellular cytomatrix

Tattym E. Shaiken, Sandra L. Grimm, Mohamad Siam, Amanda Williams, Abdol-Hossein Rezaeian, Daniel Kraushaar, Emily Ricco, Matthew J. Robertson, Cristian Coarfa, Antrix Jain, Anna Malovannaya, Fabio Stossi, Antone R. Opekun, Alyssa P. Price, and Julien Dubrulle

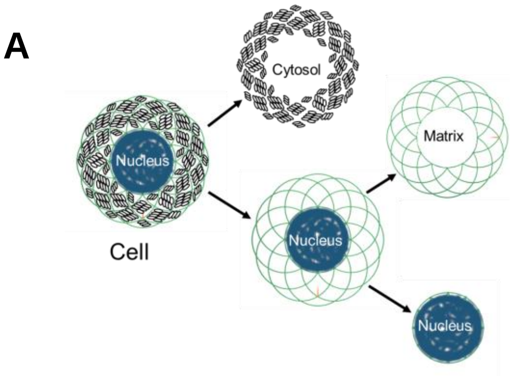

B

Ion	Relative Radius (R_i)	Molar Concentration (C_r)	R_ixC_r	Tonicity, %	Equation
Li^+	90	0.200	18	0.85	R_i x C_r = 18
Na^+	116	0.154	18	0.90	C_r = 18/R_i
K^+	152	0.120	18	0.89	

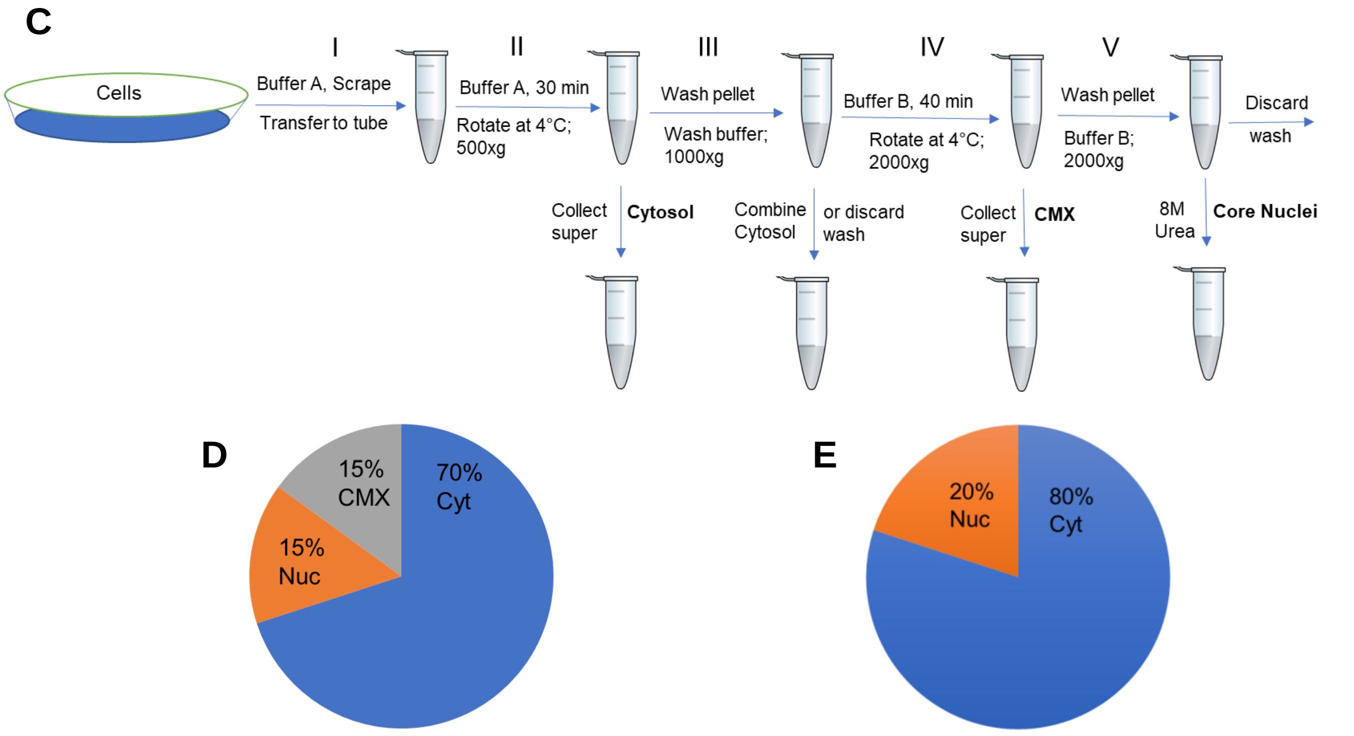

Figure S1. Cellular fractionation and isolation of the cytomatrix, related to Figure 1. (A) Schematic depiction of cell fractionation. **(B)** Equation for ion concentration calculation. **(C)** Diagram illustrating biochemical fractionation technique in a series of steps: I – washing cells with cold PBS, adding Buffer A, scraping cells, and transferring lysate to tubes; II – Incubation in Buffer A for 30 min in 4°C and collection of the supernatant as the cytosolic fraction; III – washing pellet with wash buffer (Buffer A no detergent); IV- Incubation in Buffer B for 40 min in 4°C and collection of the supernatant as a CMX; V – washing pellet with buffer B and dissolving it in 8M Urea for obtaining the core nuclear fraction. **(D)** Distribution of total cellular proteins in the cytosol (blue), CMX (gray), and nucleus (red). **(E)** Ratio of proteins in the cytosol and nuclear fractions extracted by cell lysis method. Cells were fractionated, and the total proteins were measured in each fraction to calculate the ratio. The protein percentage for each region was determined by dividing the protein found in each fraction by the total protein.

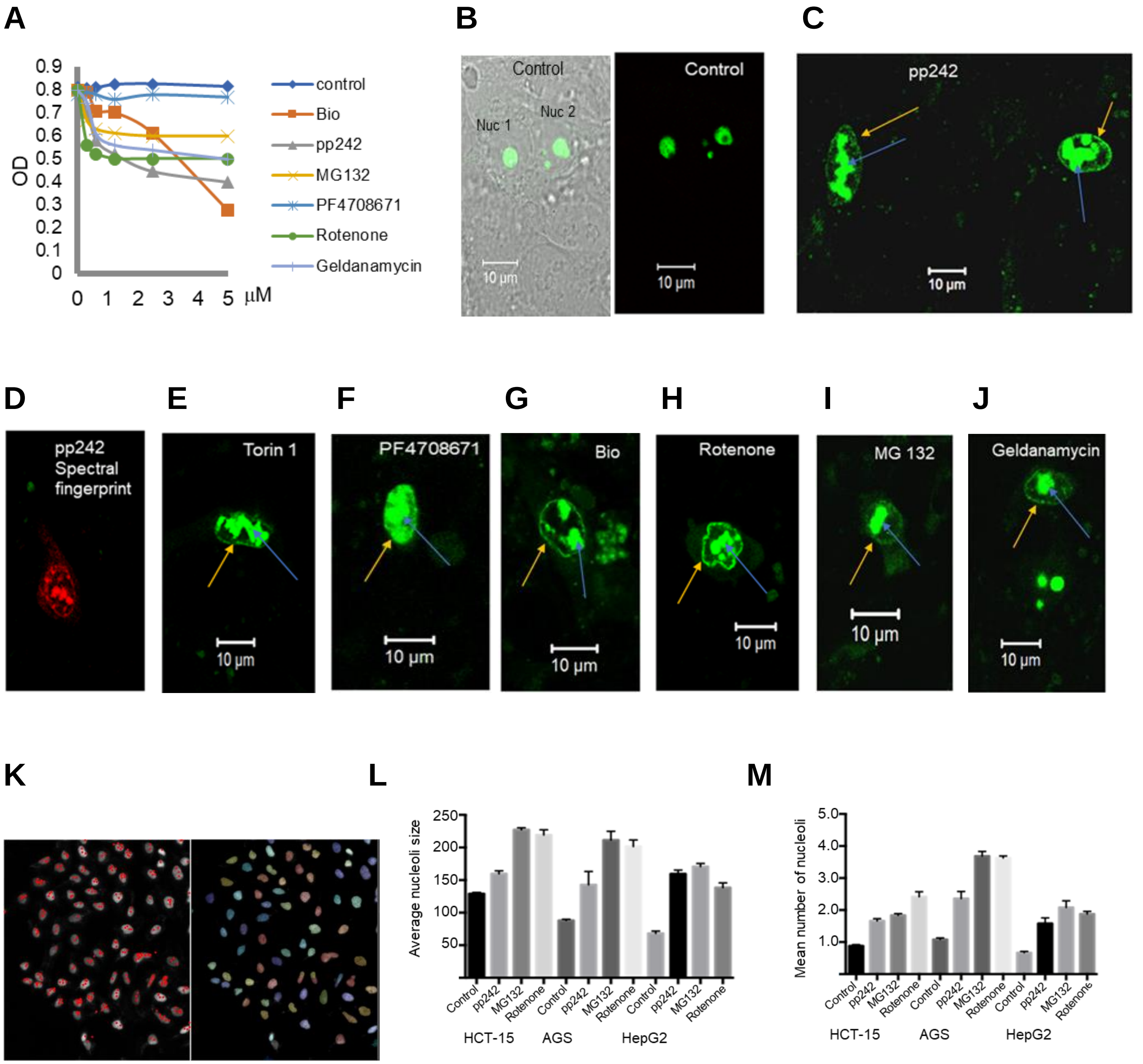

Figure S2. Effect of different compounds on HCT-15 cell growth and ribosomal L7a localization, related to Figure 2. **(A)** MTT cell growth test. **(B-J)** A live cell confocal microscopy image of cells transfected with the GFP-L7a vector pCMV6-AC-GFP. Perinuclear protein accumulation (yellow arrows) and nucleolar hypertrophy (blue arrows) were observed post drug treatment. **(B)** no treatment. Inhibitors: **(C)** mTOR, pp242, **(D)** spectral fingerprinting of rpL7a at 510 nm (for pp242) is a uniform spectral line resulting from light's emission in a narrow frequency range, compared with the nearby frequencies used to identify molecules. **(E)** mTOR, Torin 1, **(F)** RSK, PF-4708671, **(G)** GSK-3, Bio, **(H)** Rotenone, mitochondrial electron transport, **(I)** 26S proteasome, MG132, **(J)** Hsp90, Geldanamycin. **(K – M)** High-throughput nucleolar hypertrophy test in 384-well plates. **(K)** Using MatLab algorithm HCT-15 cell nuclei were highlighted to identify the size and number of nucleoli. **(L)** Average nucleolar size. **(M)** Mean number of nucleoli per nucleus. At least 200 cell nuclei were analyzed with four repeats. Data are given as means ± SD, p<0.001. Treatment time was 5 hours. HCT-15 cells were imaged on an IC200 high-throughput image cytometer (VALA Sciences). Image analysis was performed using a custom-made algorithm developed in MatLab.

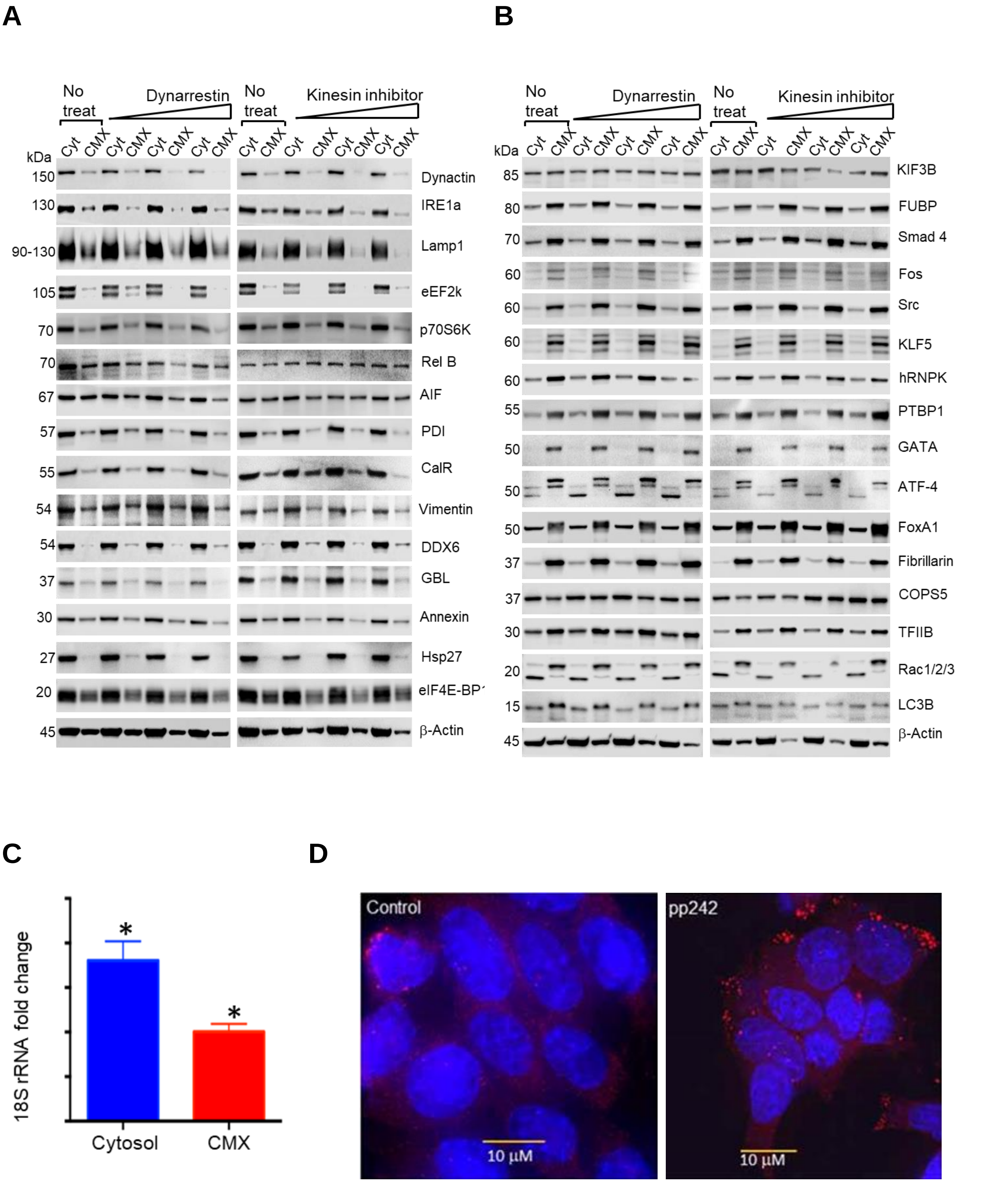

Figure S3. Analysis of protein synthesis in the HCT-15 cell compartments, related to Figure 2. (A, B) Effects of kinesin and dynein inhibitors. **(A)** Cytosolic, **(B)** Cytomatrix or mixed proteins. Effects of both inhibitors are minimal. (C, D) RT-PCR of 18S rRNA and S6 mRNA FISH. **(C)** RT-PCR of ribosomal large subunit 18SRNA; Data represent the mean ±SD (n = 3). *p < 0.05. **(D)** FISH images of ribosomal S6 mRNA in normal growth condition and pp242 treated cells.

A

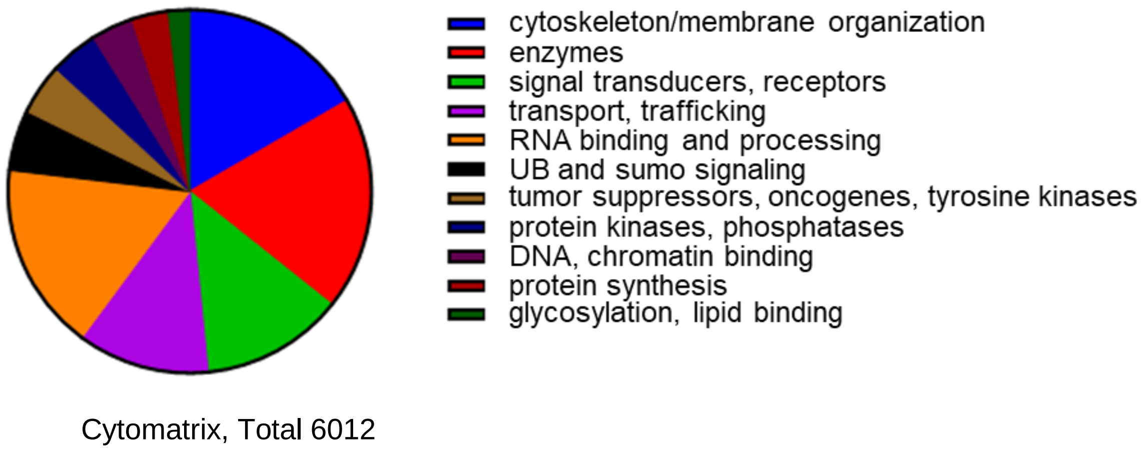

B

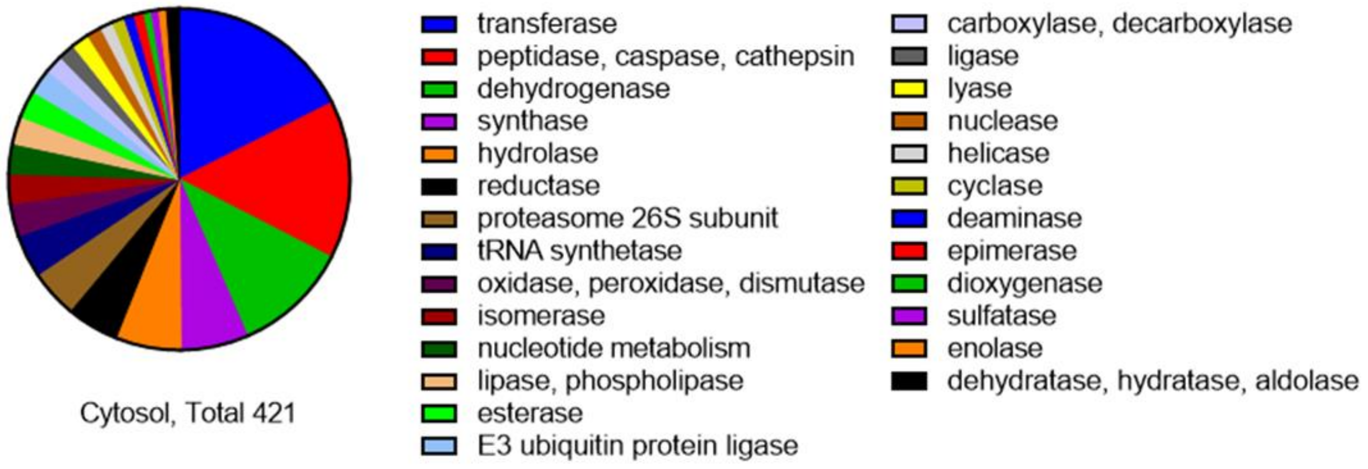

Figure S4. Pie charts of the HCT-15 cell proteome, related to Figure 3. (A) CMX proteome distribution categorized by function. **(B)** The cytosolic enzyme spectrum.

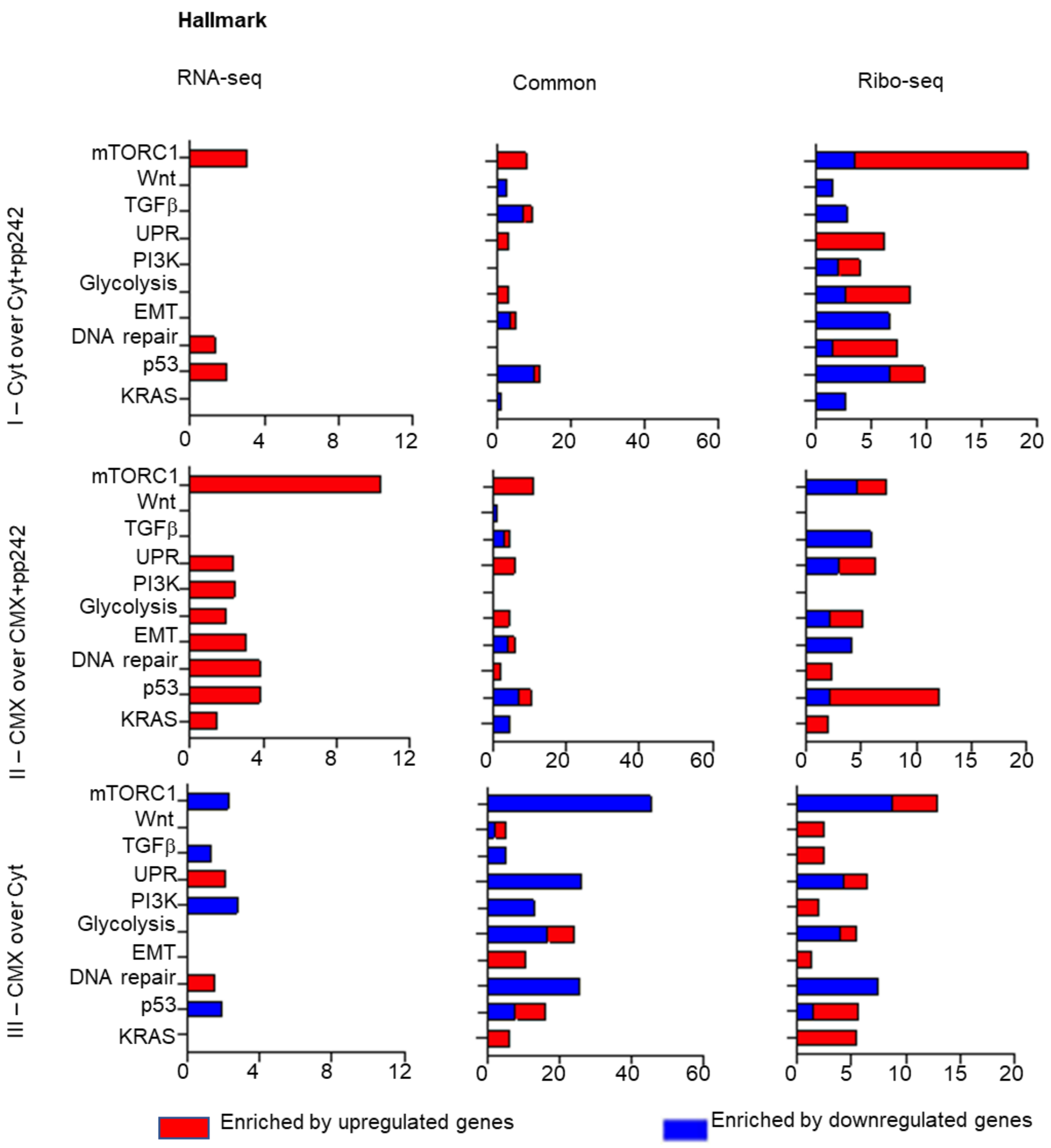

Figure S5. ORA analysis of transcription and translation of HCT-15 cells, related to Figure 4. Hallmark pathway visualization of the enriched upregulated and downregulated differentially expressed gene sets.

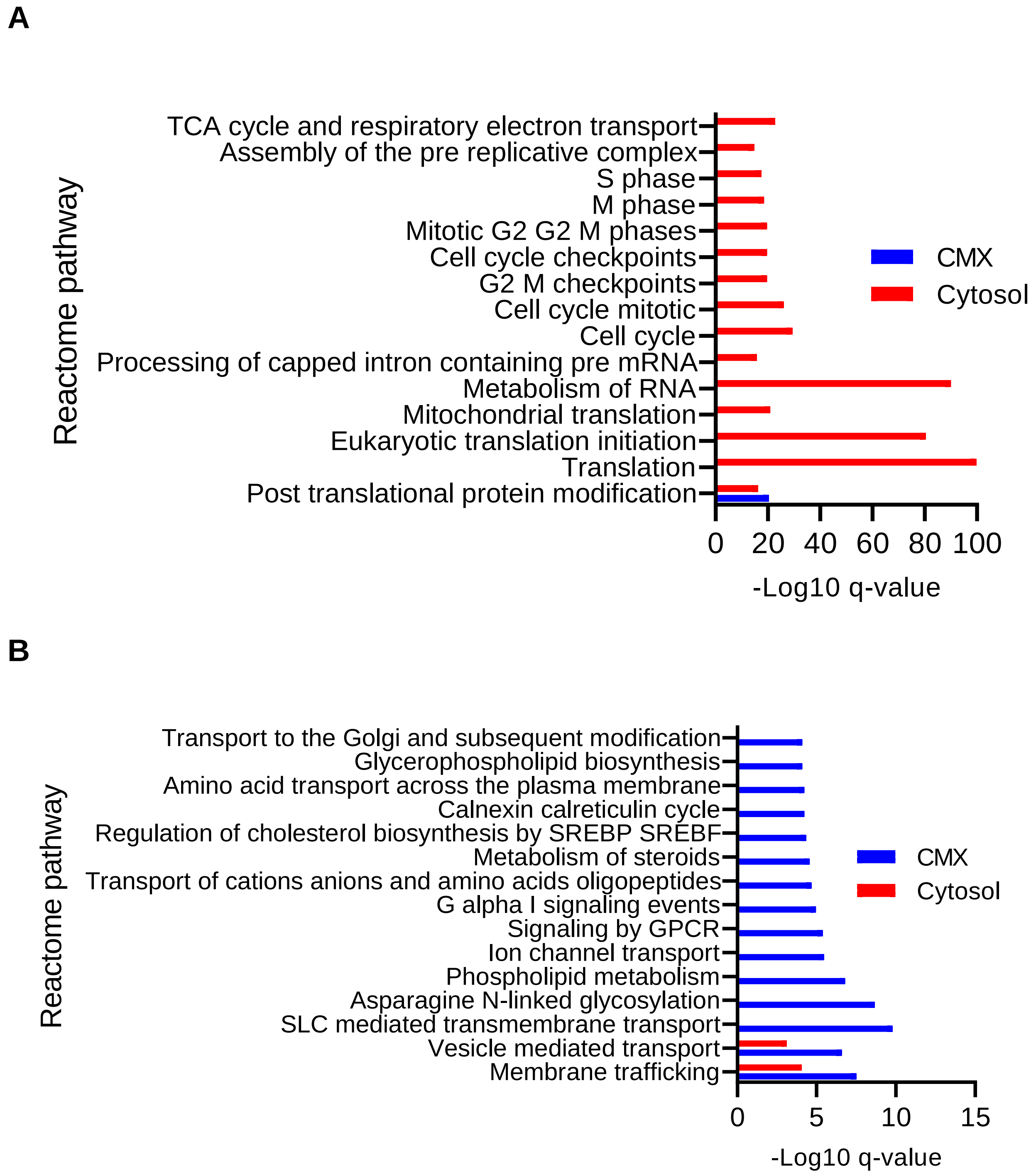

Figure S6. Reactome pathway analysis of HCT-15 cells, related to Figure 4.

Upregulated pathways were identified through ribosome footprint. **(A)** cytosol; **(B)** cytomatrix.

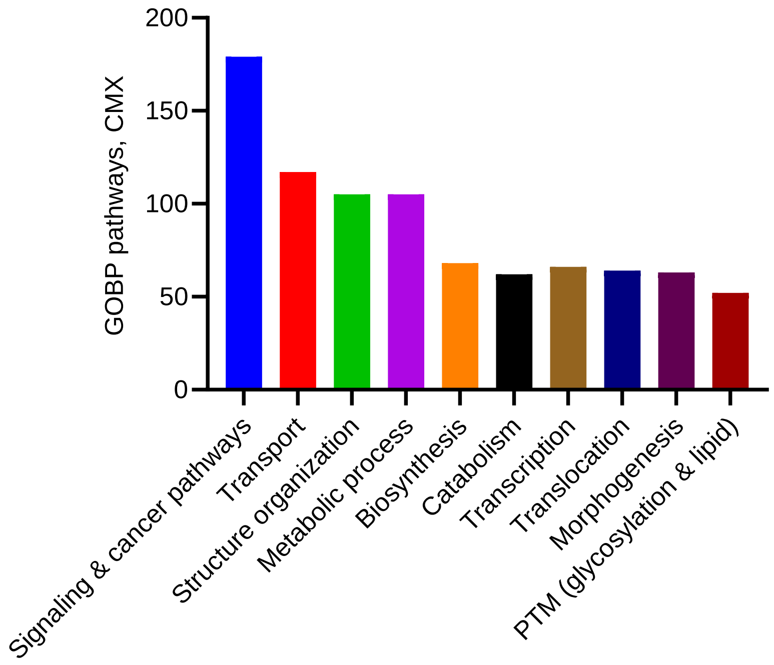

Figure S7. GOBP pathway analysis of HCT-15 cells, related to Figure 5.

Numbers of upregulated specific pathways in CMX.

Table S8. Reactome Pathway differences, related to Figure 4

Upregulated Pathways CMX over Cytosol	-log10(pValue)	
**CMX Pathway Name**	Ribo-seq	RNA-seq
SLC mediated transmembrane transport	9.80	1.81
Asparagine N-linked glycosylation	8.68	2.53
Membrane trafficking	7.50	2.44
Signaling by receptor tyrosine kinases	7.26	
Phospholipid metabolism	6.80	
Vesicle mediated transport	6.59	2.01
Ion channel transport	5.47	
Signaling by GPCR	5.39	
GPCR ligand binding	5.08	
Transport of cations anions and amino acids oligopeptides	4.67	
Class 1 rhodopsin like receptors	4.67	
Metabolism of steroids	4.56	
Regulation of cholesterol biosynthesis by SREBP SREBF	4.34	
Intracellular signaling by second messengers	4.26	
Calnexin/calreticulin cycle	4.23	
Amino acid transport across the plasma membrane	4.22	
Glycerophospholipid biosynthesis	4.10	
Transport to the Golgi and subsequent modification	4.10	
Activation of gene expression by SREBF SREBP	4.08	
Rho GTPase cycle	4.05	
Regulation of lipid metabolism by PPAR-alpha	4.05	
N-glycan trimming in the ER and calnexin/calreticulin cycle	4.05	
Sphingolipid metabolism	3.94	
Glycosaminoglycan metabolism	3.86	
Sphingolipid de novo biosynthesis	3.86	

Table S9. Reactome Pathway differences, related to Figure 4

Upregulated Pathways CMX over CMX pp242	-log10(pValue)	
**CMX Pathway Name**	Ribo-seq	RNA-seq
Respiratory electron transport ATP synthesis by chemiosmotic coupling	8.44	
The citric acid TCA cycle and respiratory electron transport	7.45	
Mitochondrial translation	7.38	
Respiratory electron transport	6.22	
Translation	6.05	
Metabolism of RNA	5.87	
Cell cycle	5.81	
DNA repair	5.33	
Signaling by rho GTPases	5.32	
Telomere maintenance	4.91	
Protein localization	4.72	
Chromosome maintenance	4.72	
Inhibition of DNA recombination at telomere	4.53	
mRNA splicing	4.31	
Cell cycle mitotic	4.31	
HDACs deacetylate histones	4.24	
M phase	4.04	
RNA polymerase I transcription	3.99	
G2/M checkpoints	3.70	
Base excision repair ap site formation	3.63	
Amyloid fiber formation	3.57	
Processing of DNA double strand break ends	3.54	
Homology directed repair	3.42	
Formation of the beta catenin TCF transactivating complex	3.29	
Rho GTPases activate PKNS	3.21	

Table S10. Reactome Pathway differences, related to Figure 4

**Upregulated Pathways Cytosol over Cytosol pp242**	**-log10(pValue)**	
**Cytosol Pathway Name**	Ribo-seq	RNA-seq
Translation	99.87	
Metabolism of RNA	90.10	1.57
Eukaryotic translation initiation	80.38	
rRNA processing	71.99	
Regulation of expression of Slits and Robos	70.09	
Eukaryotic translation elongation	67.14	
Response of eIF2ak4 GCN2 to amino acid deficiency	65.56	
Signaling by Robo receptors	65.48	
SRP dependent co-translational protein targeting to membrane	63.29	
Cellular responses to external stimuli	61.81	
Metabolism of amino acids and derivatives	57.67	
Nonsense mediated decay NMD	57.67	
Activation of the mRNA upon binding of the cap binding complex	31.59	
Cell cycle	29.44	1.31
Cell cycle mitotic	26.10	
The citric acid TCA cycle and respiratory electron transport	22.83	
Mitochondrial translation	20.94	
G2-M checkpoints	19.72	
Cell cycle checkpoints	19.65	
Mitotic G2-G2/M phases	19.65	
DNA replication	18.97	
M phase	18.54	
Respiratory electron transport ATP synthesis by chemiosmotic coupling	18.30	
DNA replication preinitiation	18.03	
DNA repair	18.03	

Table S11. Reactome Pathway differences CMX pp242 and CMX, related to Figure 4

Ribo-seq Unique Upregulated Pathways	-log10(pValue)	
**CMX Pathway Name**	CMX pp242	CMX
SLC mediated transmembrane transport	2.50	9.80
Asparagine N-linked glycosylation		8.68
Membrane trafficking	2.47	7.50
Signaling by receptor tyrosine kinases	2.26	7.26
Vesicle mediated transport	1.90	6.59
Ion channel transport	2.93	5.47
Signaling by GPCR		5.39
GPCR ligand binding		5.08
Gα–I-signaling events		4.95
Transport of inorganic cations anions and amino acids oligopeptides		4.67
Metabolism of steroids		4.56
Regulation of cholesterol biosynthesis by SREBP SREBF		4.34
Calnexin/calreticulin cycle		4.23
Amino acid transport across the plasma membrane		4.22
Transport to the Golgi and subsequent modification		4.10
Activation of gene expression by SREBF SREBP		4.08
Regulation of lipid metabolism by PPARa		4.05
N-glycan trimming in the ER and calnexin calreticulin cycle		4.05
Glycosaminoglycan metabolism	1.34	3.86
Sphingolipid de novo biosynthesis		3.86
Ion homeostasis		3.83
COPII mediated vesicle transport		3.48
O-linked glycosylation of mucins		3.26
ER quality control compartment ERQC		3.20
ER to Golgi anterograde transport		3.11

Table S12. GOBP Pathway differences and overlaps of the cytosol and CMX, related to Figure 4

**Ribo-seq Unique Upregulated Pathways, relative CMX**	**-log10(pValue)**		
**Pathway Name**	CMX/CMX pp242	CMX/Cyt	Cyt/Cyt pp242
Lipid metabolic process	17.16	30.78	5.95
Transmembrane transport	22.56	30.58	5.56
Cell-cell signaling	10.07	29.04	5.87
Cellular lipid metabolic process	18.70	28.93	5.03
Lipid biosynthetic process	12.55	21.28	1.58
Regulation of transport	17.79	21.25	5.28
Glycoprotein metabolic process	3.64	20.43	
Regulation of developmental process	11.02	18.65	3.64
Negative regulation of signaling	11.12	17.82	4.33
Cation transport	13.85	17.65	
Glycoprotein biosynthetic process	3.51	17.47	
Cell morphogenesis	14.26	15.76	3.03
Receptor protein signaling pathway	7.13	15.74	
Chemical homeostasis	11.02	15.57	
Actin filament-based process	5.58	15.55	1.69
Cation transmembrane transport	11.47	15.34	1.42
Cell migration	6.80	14.86	
Glycosylation	3.68	14.69	
Glycerolipid metabolic process	9.20	14.48	2.26
Cellular component morphogenesis	11.90	14.18	2.27
Phospholipid metabolic process	10.18	13.52	2.43
Regulation of cellular component movement	4.11	13.41	
Negative regulation of developmental process	4.47	13.22	
Lipid localization	5.03	13.08	
Cell part morphogenesis	10.71	12.75	1.95

Table S13. GOBP Pathway differences and overlaps of the cytosol and CMX, related to Figure 4

**Ribo-seq Unique Upregulated Pathways, relative to cytosol**	**-log10(pValue)**		
**Pathway Name**	CMX/CMX pp242	CMX/Cyt	Cyt/Cyt pp242
mRNA catabolic process	2.45		68.78
ncRNA metabolic process			62.77
Co-translational protein targeting to membrane	1.33	2.99	59.98
Protein localization to endoplasmic reticulum		2.51	55.64
mRNA catabolic process nonsense mediated decay	1.39	3.40	54.43
Nuclear transcribed mRNA catabolic process	2.80	4.19	50.56
Protein localization to endoplasmic reticulum	1.74	3.66	48.86
ncRNA processing			42.96
Ribosome biogenesis			36.24
Endopeptidase activity involved in apoptotic process	1.43		36.18
Ribonucleoprotein complex subunit organization			34.84
Protein modification by ubiquitination			32.14
RNA processing			31.85
Cell cycle G2/M phase transition	3.00		30.92
rRNA metabolic process			29.97
Nucleoside triphosphate biosynthetic process	5.31		21.19
Nucleosome assembly	4.36		19.46
Spliceosomal complex assembly			18.59
Fatty acyl Co-A biosynthetic process			16.34
tRNA pseudouridine synthesis			15.25
Inner mitochondrial membrane organization	4.41		13.97
Negative regulation of steroid metabolic process		1.51	13.94
Nucleoside metabolic process	2.55		13.89
Microtubule polymerization or depolymerization	3.84	1.42	13.85
Protein ADP ribosylation			13.52

Table S14. GOBP Ribo-seq and RNA-seq Pathway differences and overlaps, related to Figure 4

**Upregulated Pathways**	**-log10(pValue)**					
**Pathway Name**	CMX/CMXpp242		CMX/Cyt		Cyt/Cytpp242	
	Ribo-seq	RNA-seq	Ribo-seq	RNA-seq	Ribo-seq	RNA-seq
Cellular macromolecule localization	30.99	3.88	24.59	8.68	58.68	
Cell projection organization	27.46	4.24	15.98	4.94	6.78	1.67
Establishment of protein localization	27.16	6.77	29.04	8.44	51.78	
Transmembrane transport	22.56	10.19	30.58	5.48	5.56	4.83
Protein containing complex subunit organization	22.19	4.28	11.63	5.41	67.29	
Carbohydrate derivative metabolic process	20.87	8.92	20.85	5.88	14.41	
Cellular lipid metabolic process	18.70	4.12	28.93	6.26	5.03	
Intracellular transport	17.79	1.56	30.03	9.55	51.17	
Regulation of transport	17.79	8.86	21.25	3.99	5.28	2.43
Lipid metabolic process	17.16	5.75	30.78	7.50	5.95	
Regulation of ion transport	16.48	8.45	16.24	3.68	5.34	1.75
Protein localization to membrane	14.90	2.39	14.44		37.67	
Intracellular protein transport	14.87	2.05	17.06	8.44	47.35	
Cell morphogenesis	14.26	5.30	15.76		3.03	
Cation transport	13.85	4.82	17.65	1.75		2.43
Regulation of protein localization	13.36	3.95	9.80	1.36	9.78	
Membrane organization	13.33	2.18	11.12		5.91	
Cellular response to DNA damage stimulus	13.10	5.83	2.03	6.39	36.09	2.91
Lipid biosynthetic process	12.55	4.97	21.28	1.87	1.58	
Positive regulation of ion transport	12.53	5.02	8.11	2.93	1.71	
Organelle assembly	12.51	1.35	7.42	8.06	16.28	
Protein localization to organelle	12.43		6.75	4.99	56.50	
Cytoskeleton organization	12.33	3.01	13.89	2.74	8.03	
Cellular amide metabolic process	12.04	2.82	2.99	5.97	73.78	1.63
Carbohydrate derivative biosynthetic process	11.90	6.45	14.54	3.34	6.47	
